# Sexuality in male partners of women with fibromyalgia syndrome: A qualitative study

**DOI:** 10.1371/journal.pone.0224990

**Published:** 2019-11-27

**Authors:** Patricia Romero-Alcalá, José Manuel Hernández-Padilla, Cayetano Fernández-Sola, María del Rosario Coín-Pérez-Carrasco, Carmen Ramos-Rodríguez, María Dolores Ruiz-Fernández, José Granero-Molina

**Affiliations:** 1 Faculty of Health Sciences, University of Almería, Almería, Spain; 2 Department of Nursing, Physiotherapy and Medicine, University of Almería, Almería, Spain; 3 Adult, Child and Midwifery Department, School of Health and Education, Middlesex University, London, United Kingdom; 4 Faculty of Health Sciences, Universidad Autónoma de Chile, Temuco, Chile; 5 Clinical Pharmacy, Málaga, Spain; 6 Fibromyalgia Association of Almería, Almería, Spain; University of Würzburg, GERMANY

## Abstract

The aim of our study was to understand how male partners of women diagnosed with fibromyalgia syndrome perceive sexuality. Gadamerian hermeneutic phenomenology and the Roy Adaptation Model provided the overall framework for this research study. Eighteen participants were recruited through convenience and purposive sampling. Data collection was conducted between February and July of 2017 and included a focus group and twelve in-depth interviews. Two main themes were extracted: "facing a new sex life" and "resisting the loss of the couple’s sexuality". Fibromyalgia syndrome compromises the couple’s sex life. Enhancing intimacy, skin-to-skin contact (during acute FMS outbreaks), finding new positions, non-coital sex and use of sex toys can increase female desire and help coping.

## Introduction

Fibromyalgia syndrome (FMS) is a non-joint rheumatic syndrome that presents patients with chronic musculoskeletal pain and painful points sensitive to body pressure [1, 2]. Fibromyalgia syndrome includes fatigue and muscle pain, difficulty sleeping and stiffness upon awakening [3,4]. Fibromyalgia syndrome is associated with psychiatric comorbidity such as depression, anxiety, emotional stress and coping problems [5–8]. Fibromyalgia syndrome is a chronic musculoskeletal disease which affects physical, mental and sexual health [9], FMS affects 2.7% of the global population [3] and 2.4% of the Spanish population. Although FMS is not associated with age or gender [10], 80–90% of patients are middle-aged women who are nearing menopause [11, 12]. The criteria to diagnose fibromyalgia is to have suffered generalised pain for at least 3 months as well as pain on digital palpation in 11 out of 18 tender point sites [13, 14]. Other symptoms such as diffuse muscle pain, sleep disorder, mood disorder, headaches, fatigue and rigidity can also inform the diagnosis [15–17]. The approach to FMS is multifactorial [11]. Treatment combines analgesics, anti-inflammatory drugs, corticosteroids and psychotropic medication; however, the effect of most of them is moderate [18, 19].

Fibromyalgia syndrome is more prevalent in women and it affects their physical, psychological and sexual health [15]. Fibromyalgia syndrome is associated with dyspareunia, little vaginal lubrication, loss of desire and difficulty to reach orgasms [7,12, 20]. Along with bodily and genital pain, anguish, fear, loss of self-esteem and decrease of sexual relations lead to FSD [12, 21, 22]. Fibromyalgia syndrome affects female sexual function [23]; desire, arousal, orgasm, lubrication and satisfaction are lacking in patients with this disorder as compared to healthy controls [24]. Fibromyalgia syndrome is associated with female sexual dysfunction (FSD) [9, 20] and sexual disorders in relationships [25]. Confronting FSD in FMS requires social and partner support [26]. Women with FMS are supported by their partners in trying to understand the symptoms, seeking information and dealing with the condition, taking responsibility for home duties and childcare [27–29].

According to the Sexual Adaptation Model, people and family are complex adaptive systems that are exposed to focal, contextual and residual stimuli [30]. Fibromyalgia syndrome operates as a focal stimulus that activates a "sexuality coping process", with adaptive responses on physiological, self-esteem, role function and interdependence levels. Partners of women with FMS operate as a contextual stimulus that can contribute to this coping process. A couple’s sexuality is deeply affected by FMS [31]. Although the experiences of women with FMS have been studied [22], research on their partners’ experience is lacking. The aim of this study was to understand how male partners of women diagnosed with fibromyalgia syndrome perceive their sexuality.

## Methods

### Study design

This is a qualitative and interpretative study based on Gadamerian hermeneutical phenomenology [32]. For Gadamer, understanding a phenomenon implies a process of interpretation that is mediated by our pre-understanding, culture, tradition and history.

### Recruitment of study participants

**Fig 1:**
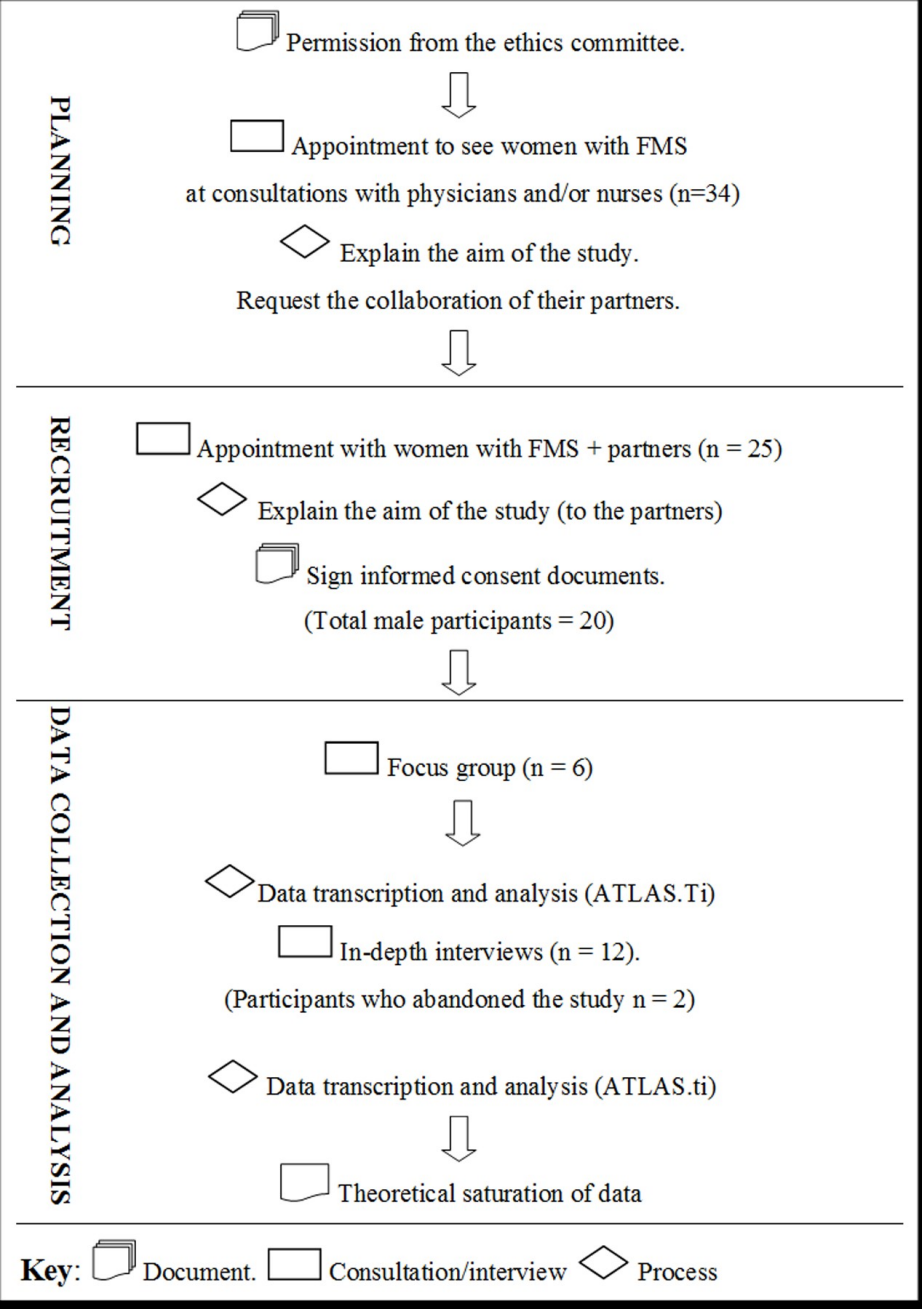
Recruitment of study participants. Eighteen participants comprised the final sample as two abandoned the data collection for professional and personal reasons (Table 1).

Through convenience and purposive sampling, male partners of women diagnosed with FMS were selected. Inclusion criteria were: to be a man ≥ 18 years old, to have been the sexual partner of a woman diagnosed with FMS ≥ 6 months and to agree to participate in the study. The exclusion criteria were: to refuse to participate in the study and to have been diagnosed with FMS. General practitioners and nurses who treat women diagnosed with FMS facilitated access to the participants. After obtaining permission from the ethics committee, the researchers contacted 34 women with FMS during consultations with doctors and nurses, where they asked for their cooperation in contacting their partners. A total of 25 women and partners went in for a visit. The aim of the study was explained and their participation was requested. Twenty male partners of women with FMS agreed to participate by signing an informed consent document. Subsequently, each partner was scheduled to participate in a focus group or an in-depth interview (Fig 1).

**Table 1:** Socio-demographic data of the participants (N = 18).

Participant	Sex	Age *M* = 50.1 SD = 8.7	Profession	Associated pathology	Years as a couple *M* = 23.1 SD = 13.1	Time with FMS *M* = 3.7 SD = 2.8
FGM1	Male	53	Teacher	No	26	10 months
FGM2	Male	45	Banker	No	10	1 year
FGM3	Male	48	Taxi Driver	Arthritis	27	5 years
FGM4	Male	56	Waiter	No	30	4 years
FGM5	Male	42	Public Worker	Atherosclerosis	15	2 years
FGM6	Male	39	Delivery Man	Type 1 diabetes	6	8 months
DIM1	Male	50	Mechanic	No	28	3 years
DIM2	Male	51	Psychologist	No	9	3.5 years
DIM3	Male	67	Retired	Arterial hypertension	53	9 years
DIM4	Male	47	P.E. Teacher	Arterial hypertension	13	2 years
DIM5	Male	48	Builder	No	11	1 year
DIM6	Male	53	Bank Manager	No	32	10 years
DIM7	Male	68	Builder	Osteoarthritis	46	7 years
DIM8	Male	45	Agriculturist	Obesity	25	5 years
DIM9	Male	46	Public worker	No	18	2 years
DIM10	Male	65	Retired	Arterial hypertension	41	7 years
DIM11	Male	43	Businessman	No	13	4 years
DIM12	Male	37	Professor	No	14	1 years

FGM = focus group man. DIM = in-depth interview man. *M* = mean. SD = standard deviation.

### Data collection

The study took place in a psychological care centre and a FMS patient association. Data collection included a focus group (FG) and in-depth interviews (IDI) and it was conducted between February and July of 2017. Six men participated in FGs and twelve participated in IDIs. Pre-understanding is central in Gadamerian hermeneutic phenomenology. FGs and IDIs were conducted by a psychologist who is an expert in sexology with four years of experience in working with women with FMS. First, a FG was carried out in a psychological care centre. It lasted 45 minutes and began with the question: "Can you tell us what the words fibromyalgia and sexuality suggest?" The IDIs were carried out after the FG at an FMS patient association, which lasted 35 minutes on average. The IDIs addressed questions that had not been clarified or that FG participants did not want to answer in public. The FG and IDIs ended with the question: "Do you want to say anything else on the subject?" (S1 and S2 Tables). Data collection stopped when recurrent patterns became evident in the participants’ narrations and data saturation was reached. The FG and IDIs were recorded, transcribed and incorporated into a hermeneutic unit that was later analysed with the aid of the ATLAS.ti 8.0 software.

### Data analysis

According to our philosophical framework, Fleming’s model was used for the data analysis [33]. In the first step, the authors affirmatively answered the question "Can the perception of sexuality in male partners of women with FMS be studied from the hermeneutical phenomenology?” In the second step, the researchers reflected upon their pre-understanding of female sexuality and FMS, which derived from previous research and clinical experience. In the third step, the researchers sought to understand the phenomenon speaking to the participants. The FG and IDIs allowed the researchers to delve deeper into the experiences of partners of women with FMS and led to new questions such as: *What sexology advice do women with FMS need*? In the fourth step, the researchers sought to understand the phenomenon through a dialogue with the text. After reading and re-reading the transcripts of the FG and IDIs, the participants’ experiences were examined and new questions emerged: *What interventions could improve the couple’s sex life*? The transcripts were analysed line by line and quotes, codes, themes and sub-themes were extracted. Three members of the research team independently extracted codes, and any discrepancies found were resolved through consensus. If the researchers did not agree on a particular unit of meaning, theme or sub-theme, this was excluded from the analysis and a more appropriate solution was found. In the fifth stage, the reliability and rigour of the qualitative data were established. An independent researcher verified that all the participants’ perspectives had been included and the participants agreed with the topics and sub-themes extracted by the researchers. Recordings, data analysis and interviews were saved to guarantee dependability.

### Ethical considerations

The participants were informed about the purpose of the study and their voluntary participation, and they signed informed consent documents. Permission was requested to record the conversations, guaranteeing access to the results. Anonymity was preserved by coding the interviews. Approval was obtained from the Ethics and Research Committee of the Department of Nursing, Physiotherapy and Medicine at the University of Almería (N°: 22/2017).

## Results

The final sample was comprised of eighteen participants, none of whom reported any traumatic experiences or sexual abuse in their partners’ childhood (the women with FMS). After analysing the data, two main themes were extracted and they may explain the perception of sexuality in male partners of women diagnosed with FMS (Table 2).

**Table 2:** Themes, sub-themes and units of meaning.

Themes	Sub-themes	Units of Meaning
Coping with new sexuality.	Fibromyalgia syndrome as an obstacle to the couple’s sex life.	Decrease of sexual encounters, lack of lubrication, lack of variety in positions, fear of hurting them, impact on relationship, comparison with other stages.
	Reconciling different levels of desire.	Loss of desire (in the woman), understanding, pain and medication that diminish desire, when caresses hurt, frustration, male desire diminishes.
	When the couple's relationship is beyond sex.	He sees her as beautiful, he still desires her, FMS does not end a solid relationship, giving up the previous sex life, hope for improvement and reappearance of desire, abandoning their partner is not an option.
Resisting the loss of the couple’s sexuality	Forbidden to surrender: imagination is power.	Alternatives to penetration, encouraging the recovery of desire, rediscovering seduction, patience and imagination, toys, movies and massages, understanding about rejection.
	Asking for sexology support.	Getting involved in the search for support, break taboos on sexuality, role of patient associations, resorting to masturbation, thinking about prostitution, the relationship beyond sex.

FMS = fibromyalgia syndrome.

### Coping with new sexuality

Fibromyalgia syndrome interferes in the development of satisfactory sexual activity in women. Constant pain, stiffness and fatigue contribute to diminished desire. In addition, the side effects of the medication interfere and complicate sexual encounters. Women with FMS and their partners face changes that affect their sexuality.

### Fibromyalgia syndrome as an obstacle to the couple's sex life

Our participants confirm that FMS brings multiple changes to their sex life. The frequency of sexual relations progressively decreases until it almost disappears. The way sexual relations take place also changes and they become restrained and predictable. The situation results in a loss of spontaneity in male sexuality, which is inhibited by fear and caution in order not to harm the woman. These changes severely affect the relationship and requires awareness of both members in favour of readjustment.

*Of course it interferes*, *everything changes*. *From being an active couple to* … *almost lacking a sex life*. (DIM4)

The participants report that their partners (the women) link the decline in sexual intercourse with a lack of lubrication. Nonetheless, they associate this problem with age, lack of desire linked to the pharmacological treatment’s (antidepressants) side effects and painful or negative experiences during previous sexual encounters. The lack of lubrication produces pain and discomfort during sexual intercourse, which affects both women with FMS and their partners. This is how one participant explains it:

*She says that she is very dry inside*, *that she is afraid because it hurts a lot*. *And it's true*, *I notice it too*, *it's very annoying* … (DIM4)

Men become inhibited in their sexual encounters; they are fearful of trying new positions or movements that may cause pain or discomfort to women. Faced with this situation, the male partners of women with FMS gradually incorporate alternative practices such as massages, movies, erotic reading or the use of sex toys. The objective is to generate new scenarios where they can create pleasurable sexual experiences with the least negative impact on a physical level. In addition, men notice a lack of relaxation in women that affects both sexual intercourse and any other sexual practices. Our participants fear the impact of this situation on the romantic relationship. The comparison with their previous sex life becomes inevitable.

*Before*, *it was not like this*. *However*, *now*, *intercourse is not a priority*. *Sexuality can also be experienced as skin on skin*, *soul to soul*. *But for this you have to be relaxed and it is difficult with FMS*, … *sometimes impossible*. (DIM6)

### Reconciling different levels of desire

Sexual desire is severely diminished in women with FMS and their male partners understand this situation as an effect of the condition. Women’s discomfort is constant, related to the physical overexertion inherent in the sexual relationship and it can turn into pain and continue for days. Men experience ambivalent feelings caused by a combination of their sexual desire and an understanding of women’s reasons to refuse sexual encounters, especially if they have experienced lower sexual desire as a consequence of other conditions. This is how one participant explains it:

*It is hard for her to decide and I understand her because I have other pains and if you move*, *your desire diminishes*. *Well*, *for her… with FMS*, *even more so*. (FGM)

Men associate the women’s lower libido with physical factors such as discomfort, stiffness or lack of sleep. They also link it to psychological factors such as depression, anxiety or the pharmacological treatment’s side effects. Men know that long-term treatments to alleviate the FMS symptoms have a negative impact on their sex life. This is a situation that shapes their daily life as a couple and they must adapt to it:

*The medication affects her a lot*, *antidepressants lower her libido and it takes years*. *She knows it*, *she accepts it*, … *and so do I*, *we both do*. (DIM7)

Even caresses and skin-to-skin contact are severely compromised. In the stages of acute FMS outbreaks, women report discomfort with only a light touch meaning that interactions and contact with their partners almost disappear. Men feel frustrated and although they willingly give up sexual intercourse, they are reluctant to accept not having any form of sex life. This is how one participant explains it:

*A simple caress is pain*, *an advance is tension*, *friction is discomfort*, … *it takes away the desire to touch*, *to look*, *everything* … *because you do not know what her reaction will be*, *if it will bother her*, *if it hurts*! (DIM11)

The participants feel they need to be extremely careful with the way they establish physical contact with their partners and this makes their sexual desire decrease. When a sexual encounter is initiated, men start worrying about their partners’ feelings and reaction. Men start wondering whether the situation could be causing discomfort and their partner does not say so in order for them to enjoy themselves. Consequently, sexual relationships begin to be less interesting because being so aware of the woman’s reactions entails a lack of improvisation, loss of imagination and falling into monotony.

*Always doing it (intercourse) the same way/posture*, *without being calm*, *is a monotony that kills desire*. *There is no spontaneity*, *no new positions*, *me on top and little else* … *this limits our sex life a lot*, *it is frustrating*. (DIM1)

### When the couple's relationship is beyond sex

According to our participants, FMS profoundly affects the couple's sex life. However, their love for their partners and the nature of their relationship are of utmost importance. If there is a strong relationship, our participants do not fear facing a “sexuality coping process” to overcome the difficulties of FMS. The participants soon become aware that they must “reset” their relationship and they develop coping strategies such as promoting joint leisure activities in an attempt to avoid focusing their lives exclusively around FMS.

*We enjoy everyday things together like going to the movies*, *we seem to forget the FMS*, … *we are reigniting our beginning as a couple*. (DIM5)

Although sexual relations change, the participants see women as their sexual partner and they still feel sexually attracted to her. The participants try to replace intercourse with practices that have less physical impact, such as masturbation, oral sex, the use of sex toys, oils, etc. Massages are also a widely used practice that relieve pain and praise the female body, allowing them to show their dedication and desire. This contact is of great help for FMS since it enhances the physical, sexual and personal identity of the woman (they often do not perceive themselves as desirable/attractive) and it praises their self-esteem. This is how one participant explains it:

*You look beautiful*, *I'm getting in the mood (for sexual relations)*. *And she says*: *do not start now*! *She knows that when I give her the massage I get excited*, *I get aroused*, … *others (women) wish they had the body that you have*. (DIM9)

Our participants state that if the couple had a strong relationship, they will remain together. There is an adaptation process where both members must evaluate the mismatch caused by the FMS and settle on the reasons to stay together. This is a complicated process in which sexual loss is compensated with other positive aspects in the couple's life. Men encourage both practising joint activities that are not too physically-demanding (for example: taking short daily walks to fight muscle rigidity) and speaking, listening and understanding each of their partners’ needs. As a couple, they share a connection and men are willing to give up a sex life as active as they would like to have or actually had prior to FMS.

*If as a couple you have a solid base*, *like us*, … *you get along*. *Because sex does not keep me here*, *I could look for that anywhere*. (FGM)

Women are their companions and a good relationship usually balances the loss of sexual relations. Most men make a positive assessment of their life as a couple even if they had to give up an active sex life. Although this is not to their liking, they accept it as a vital setback that they must face together.

*In sexual relations we have accepted it and* … *I do without*. *I will not say it with pleasure*, *but yes*, *I have largely done without*. (DIM7)

Although some men love their partners and are sure of their feelings, they do not accept the situation. They see sexuality as a primary need that in some cases has not been met for years. The participants demand women with FMS to change their mentality and would like them to either get involved in sexual activities that do not cause them pain (for example: masturbation or oral sex), or to get their consent to satisfy their sexual needs outside the relationship.

*There is a very closed mentality and sexuality is like eating*, … *it is not so easy to give up*. *I would appreciate it if my wife told me*, *do you need sexual relations*? *I cannot find them (elsewhere)*, *it is okay*. (DIM9)

However, all of the participants hope for the FMS symptoms and the woman’s quality of life to improve, so that the couple’s sex life can also improve. This positive attitude keeps the couple together and motivates them to face the day-to-day struggles. Men’s hope in the resurgence of sexual desire could help the couple to return to their previous sex life. Men are called upon to wait, hoping that female sexual desire will come back.

*When she gets better*, *it will change*, *because we have not lost hope that the FMS will improve*, *and then there will be more demand*, *a resurgence*, *right*? (DIM1)

They all agree that they would not be better alone, separated, or with another woman. For our participants, their partners are more than sexual partners; they are motivated to grow as human beings. They love their partners above everything else and FMS does not stop them from continuing with their life project as a couple.

*We look at each other and say*: *well*, *this is how it is*, *we have to accept it*. *When we get older we will continue being together*, … *the rest is secondary*. (DIM2)

### Resisting the loss of the couple’s sexuality

The participants do not give up; they take the lead in finding solutions for all the difficulties associated with FMS. Regarding sex, they initiate the use of a wide variety of resources that allow them to continue enjoying their sexuality. They seek to awaken female desire and to foster women’s sexual fantasies resorting to aromatherapy, music, relaxation techniques, spas or thermal baths. However, these practices require preparation, interest from both partners and information. Men try to cushion the impact of FMS on the couple's sexuality but they lack support from sexology experts.

*Yes*, *they tell us about pain*, *about medicines* … *but not of sexuality*, *nothing*. *As if it ceases to exist*, *and no*, *I have my needs*, *that does not change*! (DIM2)

### Forbidden to surrender: Imagination is power

Since intercourse is not always possible, men are forced to explore alternatives that make their sexual practices pleasurable. They create a sexual climate using music, relaxation techniques, erotic readings, cinema trips and romantic dinners. For our participants, exploring new techniques that nourish their partners’ and their own sexual desire and enjoyment is key; hence why they do not hesitate to adapt and change their sexual practices.

*It bothered her so much that penetration was impossible*. *Well* … *Touching it is*! *If I have to "have fun" more with the clitoris than with the vagina*, *then with the clitoris it is*. (DIM3)

The couples’ sex life and the way they interact with each other undergoes a drastic change. For our participants, FMS activates creativity and allows them to discover a world of unexplored possibilities while enriching the spectrum of the couple's sexuality.

Things have to change. No penetration but I know that toys and clitoris (stimulation) work very well for her, so, perfect … I will buy new toys. (DIM6)

When attempting to physically approach their partners, men feed desire through seduction by proposing new experiences and sexual fantasies. Men believe that being witty and creative helps the relationship to regain the uncertainty of wooing. Both members enjoy experiencing the delicacy of courtship as well as the uncertainty and novelty of the unknown. Sexual desire of both partners tends to be nourished by these innovative activities.

*I got a room with a jacuzzi*, … *she was already willing*. *But you have to wait*, *seek*, *convince her intelligently* … *[*…*] it has its charm that*, *at this age*, *every time you want to have sexual relations you have to woo her*. (FGM)

Men believe that they must have patience and not waver. They know that sexual desire must be nourished so the woman will be aroused and will acquiesce to the sexual encounter. Although the participants accept that they have to be persistent and always believe in the possibility of a sexual encounter materializing, they also admit that this can be tiring.

*It is about persisting*, *about waiting*, *because if she starts*, *she gets aroused*. *But I would be lying if I did not say that*, … *sometimes you get tired*. (DIM8)

The search for alternatives is an active task for the couple. Sometimes women take advantage of days in which they feel better to actively seek a sexual encounter, even resorting to toys acquired at sex-toy parties or online. Although they are aroused, they suffer from lack of lubrication. They must adapt to the use of lubricants to alleviate vaginal dryness. Men show that they have adapted to these practices in their sexual relationships.

*She has a lot of dryness*, *but we use lubricants*, *it depends on the moment*. (FGM)

Men agree that women have trouble starting but when they acquiesce to having sex, they enjoy it and have orgasms. Although sex implies physical activity and they get tired, it benefits them as it improves their mood, alleviates pain, allows them to sleep and rest more. Men state that, after having sex, their partners wake up feeling relaxed, freed from tensions and with a more positive attitude.

*Once the foreplay is done*, *she enjoys herself and has a good time* … *she orgasms eagerly*. *And even if she gets tired*, *the next day she has less pain*, *and physically looks better*. (DIM3)

If despite their attempts, wooing does not work, men tend to be understanding. They understand that this is due to the condition and they accept it with a resilient attitude. They know the difficulties their partner faces with FMS and use patience as an infallible ally in those cases.

*She's having a hard time so okay*, *if not today* … *well next week*. (DIM5)

### Asking for sexology support

Men report that their partners have not had any kind of sexology support after the diagnosis. However, they also admit to not having looked for it in the first instance because when FMS is diagnosed, sexual relations are not considered the main issue. Once the situation returns to normal, sexuality emerges as a pending issue for the couple to resume. This is the point when doubts, questions and the need for help arise.

*Sooner or later we will have to talk (to a specialist)*. *Sex is a very important part of a couple that is dying little by little*. (DIM5)

Although patient associations try to holistically help women with FMS, sexuality tends to remain in the background. The public health system does not offer any type of sexology advice to help women and their partners to confront the impact of FMS on their relationship and quality of life. As one participant says, they only consider seeking advice from a sexologist a while after the diagnosis.

*Sure*, *we'll have to do something*. *I'm waiting to see if it improves a little to address the issue*, *but if it does not change*, *we'll have to talk about it*, *ask for help …* (DIM1)

Men believe that sex counselling could help them to cope with the side effects of the condition. Patients and their partners could benefit from professional support and they consider it essential to normalise the sexuality-related difficulties that the couple will have to face during their lives.

*Yes*, *we must break taboos and find solutions for these issues [*…*] The psychologist at the association can open way by saying*, *"hey*, *it is okay*, *this is what you get*, *just as you take pills*, *you also have to look out for your partner*. (DIM10)

Our participants also highlight the role that patient associations can play. Fibromyalgia syndrome in women is associated with anxiety and depressive disorders; it is a socially stigmatised disease where the diagnosis and the testimonies of the patients are often questioned. On multiple occasions, it is the male partner who encourages women to seek help in patient associations. These women only talk about their experiences with other women with FMS, as well as those related to intimacy and sexuality. Sharing experiences with other couples could help soften the negative effects on their sex life.

*The association would have to get more involved*, *focus patients on this issue*, *lead us to professionals*, … *nobody talks about sexuality but it is there*. (DIM6)

For men, sexuality is still a primary need to address. In some cases, they do not consider it as something imperative and, although they are dissatisfied, they settle for sporadic sexual encounters with their partners. In other cases, men are forced to develop strategies to enjoy their sexuality without their partners, masturbation being one of the options they use the most.

*I deal with it*, … *you masturbate every once in a while*, *period*. *It feels like a joke that you have to act as if you were a fifteen-year-old kid*, *but it is what it is*. (DIM1)

Men refer to prostitution but do not mention its use. Despite their high levels of sexual desire and dissatisfaction, they claim to be faithful to their wives. This opinion contrasts with that of women with FMS, who live immersed in the fear of abandonment. As one participant says, this topic does not surprise them. They have thought about it and contemplated it but they have not done it. They acknowledge it in others but not in themselves.

*At least you do not have other thoughts (prostitution)*! *The ones who go off with whores are worse*, … *at least I stay in the house*! (DIM3)

The couple’s sex life and relationship is deeply affected by FMS. Men understand and progressively adapt to the situation, giving priority to being with their partners over active sexuality. In general, FMS is not a sufficient reason to end the relationship or replace it with another one.

*With time we accept the condition*, *with its sexual aspect as well*. *I would not be better off alone*, *I would not change my wife for any woman in the world*. (DIM7)

## Discussion

The aim of our study was to explore, describe and understand how male partners of women with FMS perceive their sexuality. According to our results, the association between FSD and FMS [24] has severe repercussions on a couple’s sexual life. The Sexual Adaptation Model has allowed us to study the participants’ changes and adaptive responses [30]. Women with FMS suffer constant pain, fatigue, rigidity, anxiety and depression [20,23], together with the side effects of pharmacological treatment [18], these symptoms lead to FSD and affect the couple’s sex life [22, 34]. Fibromyalgia syndrome impairs female sexual function as a consequence of pain or stiffness, vaginal dryness and discomfort during intercourse [6, 7]. Antidepressant drugs negatively affect a couple’s sexuality, although mirtazapine and moclobemide have a significantly better tolerability profile. Based on meta-analyses, the only 'strong for' therapy-based recommendation in the guidelines was exercise, together with non-pharmacological therapy and patient education [19]. Contextual stimuli such as age or menopause and sociocultural stimuli such as the sexual role of women contribute to the loss of sexual desire and the couple’s sexual activity [7]. Our results confirm that FMS acts as a focal stimulus that affects the state of the couple’s sexuality, unleashing the sexuality coping process [30]. Concurring with Granero-Molina et al. [26], male partners of women with FMS see themselves as the main source of support, but both must adapt to a different type of sex life [35, 36]. According to Bazzichi et al. [5], our participants told us that FMS limits physical contact, sex positions, spontaneity, creativity and sexual satisfaction for both partners. Male partners of women with FMS experience the frustration of losing sexual relations but they want to make the relationship work and actively look for solutions [37]. As with other chronic illnesses [38], the couple does not give up but undergoes a coping process that can generate relational growth. Multiple determinants affect sexual function in women with a chronic pathology, which has a negative impact on their relationship [39, 40]. Being aware of the challenges that FMS poses for the relationship and engaging both partners can help restore the couple’s intimacy and sex life. Sexuality is rarely discussed with professionals [41], so our participants have discovered their own solutions and comfortable positions for the woman. According to Roy [30], the self-concept mode of adaptation consists of the individuals’ feelings about their bodies. Our participants have stated that they try to reinforce the woman’s identity, self-concept and attractiveness, cultivating eroticism and nurturing desire [37]; despite their frustration, they promote creativity by seeking alternatives to intercourse, such as recreating fantasies, using toys, lubricants and/or giving massages [42]. Although FSD in FMS is related to traumatic experiences or childhood sexual abuse [43], our results do not confirm this. The men accept the limitations that FMS imposes on their sex life but they integrate it into an emotional approach with their partner [44]. Although FMS leads to a lack of trust and fear of losing a partner [22], our results contradict these fears. The influence of other pathologies, or the decline of male sexuality with age, may explain why men resign themselves to the situation and do not abandon their female partners. Fibromyalgia syndrome hardly produces changes in female body image [45], contributing to men not losing desire, however, renegotiating sexuality within the couple is unavoidable. Women with FMS do not feel supported by their professional, social or family environment [46], with the exception of their partners who accompany them to seek help [26]. In patient associations they share experiences, feel understood and uncover their sexual problems. Despite experiencing difficulties [27, 46] and putting women first, our male participants do not lose hope of regaining their sex life. Dissatisfied with their sex life, men seek information and sexology advice [38, 47]; they compensate for sexual dysfunction by strengthening intimacy [22] and fostering new sexual practices [37, 38, 41]. Women with FMS and their partners require help from sexology experts [26, 29].

### Limitations

The results may be different in a female partner of a man with FMS, more than two members with FMS, or same-sex couples. The other conditions affecting women with FMS, or the cardiovascular or musculoskeletal pathologies of the male participants, could negatively affect the couple’s sexual life. Some results may be related to age, the length of the relationship, the time at which FMS was diagnosed, or sexual expectations throughout the natural course of the couple’s relationship.

## Conclusions

Fibromyalgia syndrome has profound physical, psychological and social consequences for women, while also compromising their sex life. Fibromyalgia syndrome forces women and their partners to face a new sex life. The coping process is a difficult struggle in which the partner (man) constitutes the main source of support for women with FMS. Male partners of women with FMS understand the problem, experience the process and seek solutions with them. Men retain their sexual desire but suffer from frustration and monotony, resigning themselves to the progressive deterioration of their sex life and relationship. However, they do not give up. Instead, in the hope of resuming a satisfactory sex life, they explore all possibilities. Men lead adaptation strategies in order to increase female sexual desire, minimize their discomfort and encourage imagination/creativity in sexual encounters. Enhancing intimacy, skin-to-skin contact (during acute FMS outbreaks), finding new positions, non-coital sex and use of sex toys can increase female desire and help the couple cope. In this process, they report the need for information, advice and help from sexology experts to improve coping. Despite resorting to substitutes such as masturbation, they resist seeking sexual services such as prostitution. For the majority of men, supporting the woman as a partner prevails over the loss of quality of their sex life.

## Supporting information

S1 Table Interview guide (English). (DOCX)

S2 Table Interview guide (spanish). (DOCX)

S1 Guide Interviews (DOCX)
