# Supplementary material for: Sexuality in male partners of women with fibromyalgia syndrome: A qualitative study
Source: PLoS One. 2019 Nov 27;14(11):e0224990. doi: 10.1371/journal.pone.0224990 (PMC6880977; doi:10.1371/journal.pone.0224990)
Supplement: S1 Table — (DOCX) [file pone.0224990.s001.docx]

**Table 1:** Interview Guide (English).

| **Stage of interview** | **Subject** | **Content/Example questions** |
| --- | --- | --- |
| Introduction | Motives | 'The belief that their experience offers information which should be known by everyone. ' |
|  | Aims | 'Carry out research to make said experience and information known. ' |
| Start | General introductory question | "Can you tell us what the words fibromyalgia and sexuality suggest?"  ‘How do you understand your partner's syndrome?'  'Many patients sometimes look very changed at the physical level after starting the medication, how do you remember or have experienced this change in your partner? How has it affected her self-esteem?' |
| Development | Guide for conversation | 'How has your sex life changed since your partner has fibromyalgia?'  'How do you react if your partner does not feel like having sex or wants to stop doing it if you are having it at that time?'  'What strategies have you used to improve sexual relations? What alternatives have you found to coitus?' |
| Finish | Final question | Do you want to say anything else on the subject?' |
|  | Thanks | 'Thank them for taking the time to talk to us.  Remind them that their statements will be of great help to us. ' |
