# Supplementary material for: Sexuality in male partners of women with fibromyalgia syndrome: A qualitative study
Source: PLoS One. 2019 Nov 27;14(11):e0224990. doi: 10.1371/journal.pone.0224990 (PMC6880977; doi:10.1371/journal.pone.0224990)
Supplement: S2 Table — (DOCX) [file pone.0224990.s002.docx]

**Tabla 2:** Interview Guide (spanish).

| **Etapa de la entrevista** | **Tema** | **Contenido / Ejemplos de preguntas** |
| --- | --- | --- |
| Introducción | Motivo | Su experiencia puede ofrecer información que todos deberían conocer. |
|  | Objetivos | Se va a llevar a cabo una investigación para dar a conocer experiencias como la suya. |
| Inicio | Pregunta introductoria general | "¿Puede decirnos qué sugieren las palabras fibromialgia y sexualidad?"  '¿Cómo entiende usted la enfermedad de su pareja?  Muchas pacientes se ven a veces muy cambiadas a nivel físico después de empezar con la medicación, ¿cómo recuerda o ha vivido este cambio en su pareja? ¿Cómo ha afectado a la autoestima de su pareja? ' |
| Desarrollo | Guía de la conversación | '¿Cómo ha cambiado su vida sexual desde que su pareja padece fibromialgia?  ¿Cómo es su reacción en caso de que a su pareja no le apetezca tener relaciones sexuales o quiera parar en caso de estar manteniéndolas en ese momento?  ¿Qué estrategias han utilizado para mejorar las relaciones sexuales? ¿Qué alternativas han encontrado al coito?' |
| Final | Pregunta final | ¿Hay algo más que le gustaría decir sobre el tema? |
|  | Agradecimiento | Gracias por tomarse el tiempo de hablar con nosotros.  Su testimonio nos será de gran ayuda. |
