# Supplementary material for: Sexuality in male partners of women with fibromyalgia syndrome: A qualitative study
Source: PLoS One. 2019 Nov 27;14(11):e0224990. doi: 10.1371/journal.pone.0224990 (PMC6880977; doi:10.1371/journal.pone.0224990)
Supplement: S1 Guide Interviews — (DOCX) [file pone.0224990.s003.docx]

**SUPPORTING INFORMATION FILE**

Voz 005_sd.m4a

**¿Hace cuánto tiempo le diagnosticaron la enfermedad?**

Realmente desde que se la diagnosticaron no hace mucho, a principios de este verano. Pero según ella me contó, ya lleva padeciendo eso como desde hace más tiempo.

**Los síntomas desde hace más tiempo, ¿no?**

Sí, los síntomas de que le duele todo. Lo que pasa es que yo lo he notado mucho, mucho en los últimos meses estos, en los que era rozarle y era como si… que además se te olvida, y te sientes mal porque se te olvida y de vez en cuando le haces cualquier cosa, pero de rozar a lo mejor, ¿no? En plan cariñoso y le duele mucho.

**Hay una hipersensibilidad…**

Hay una hipersensibilidad, sobre todo con el roce.

**Y cómo afecta, por ejemplo, a un día a día, tareas del hogar, etc.**

Sí, bueno, más o menos no va muy mal, lo que pasa es que bueno, yo suelo ayudar bastante en el hogar, compartimos mucho las cosas del hogar, por las profesiones que tenemos y eso, ¿no? También, mira, tienes que ver una cosa, yo soy profesor y vivir juntos, juntos sólo ehm… Llevo cuatro años fuera ahora llevo más tiempo, pues desde junio del año pasado hasta ahora, hemos estado viviendo juntos, pero durante cuatro años hemos estado por separado.

**Claro.**

Porque yo estoy destinado en Ubrique, lo que pasa que ahora me han dejado, por conciliación familiar, estar en mi casa, este año, entonces, eso para que lo tengas en cuenta.

Nos hemos visto sobre todo los fines de semana. Entonces las vacaciones sí, de ahí sí se puede hablar algo.

Ella es una persona que es bastante sufrida, no se le nota, tiene una fortaleza interior muy grande. Es una persona que debería estar, según lo que se comenta de esto, deprimida, pero se le nota muy poco.

Aunque tiene los dolores y eso, pero se le nota poco, o sea, ella se queja de los dolores y tal, pero lo lleva bastante bien para lo que creo que, por ejemplo, lo llevaría yo.

Se hacen duras algunas veces el tema de las discusiones, por ejemplo, es bastante más sensible que una persona normal, y donde puedes discutir con otras personas, pues con ella a lo mejor, llegas antes a un punto más fuerte, ¿no?

Por ejemplo, yo algunas veces me siento mal, porque no te das cuenta, es una enfermedad que no es muy… es como un poco invisible…

**Eso dicen, claro…**

No se ve, no es como cuando tienes un miembro roto o algo de eso. Que se ve. O por ejemplo, ahora ella está muy mal y cada vez que respira se (Sonido como de respiración difícil) y eso lo notas. Pero lo otro, parece normal porque no se ve, entonces, tú te sientes un poco mal en el sentido de que muchas veces dices: “¡Coño!”.

A lo mejor, venga, vamos a andar, ella a lo mejor, no puede andar mucho, o anda muy poquito, entonces…

Claro, yo estoy más en forma, y después andamos, pues andamos 4 Km, y después está hecha polvo, entonces luego tú te sientes mal porque dices, ¡Ostias! Y ya no me he dado cuenta de que yo tendría que haber ayudado un poco, o tenía que haberme…

Entonces, ya vas constantemente diciendo: “¿Paramos ya? ¿Paramos ya?”

**Claro, porque también, quizás, intenta comprenderla…**

Claro, es intentar comprenderla. Lo que pasa es que muchas veces, a mí, por ejemplo, se me hace difícil, veo que no lo hago bien en ese sentido.

**Y qué podría hacer mejor, para…**

Ser un poco más sensible con respecto a su enfermedad, no sabría cómo hacerlo realmente... Porque algunas veces es tan débil… se te viene abajo con cosas que para ti son completamente normales, y para ella pues, a lo mejor, un esfuerzo durante 20 minutos, pero un esfuerzo, te digo, yo qué sé de estar limpiando la casa entre los dos, estar cocinando durante 20 min, y después se sienta en el sofá y está hecha polvo, literalmente hecha polvo, ¿vale? Entonces eso se hace difícil. Porque en el momento en que lo estás haciendo no eres consciente, porque te cuesta a ti tan poco, es algo tan normal…

Porque si estamos, qué te digo yo, haciendo senderismo, y subiendo una montaña que cuesta trabajo a los dos, te das cuenta, pero cuando es algo que es sencillo, que es cualquier cosa… y en ese momento no eres consciente. Y supongo que, estar más tiempo con esa enfermedad, para ir cogiendo más conciencia, e ir sabiendo cuáles son los límites de ella.

**5:11 Y la medicación, ¿ayuda?**

¿La medicación que ella toma?

Sí, sí le ayuda, lo que pasa es que toma muchísima, toma mucha. Otra cosa también, va mucho de médicos, eso también lo veo yo y también me entristece un poco por mi parte. Es que es casi todos los días de médico, es horrible, porque yo a lo mejor voy de médicos dos o tres veces al año y las dos o tres veces me está molestando (ir), a veces cuando me pondo en la situación de ella, pues, dices Joder, yo esto… esto es horrible, yo no, no sabría soportarlo, ¿no? Y también se hace difícil por lo que es tu parte, ¿no? Por ejemplo, su hija, no es mi hija, está estudiando las oposiciones y tiene que acompañarlas muchas veces, porque claro, yo por las mañanas no puedo acompañarla al médico, la tiene que acompañar ella también, y se le está haciendo muy duro porque está preparando unas oposiciones, y entonces tiene que, cuando la pobre puede, levantarse muy temprano, y a lo mejor, después, está semana está más libre pero hay semanas que es, a lo mejor es un día el que se libra de ir al médico, y la media son dos horas diarias, de estar en el médico, como media, te estoy diciendo. Dos horas diarias, es lo que echa en el médico.

**6:22 Pobre…**

**Y la medicación, le ha afectado a nivel físico…**

Mira, ella ahora, ha tenido la suerte de que le han mandado un medicamento, un internista, que es la coechicina, no sé si la conoces,

**Mmm… no**

No, no? Bueno pues eso le está haciendo mucho. Porque le detectaron una infección en la sangre, y le atacaba también a todos lados. Y eso le está ayudando mucho, mucho. Entonces llevamos como dos semanas, y ya la veo yo con más ganas.

Por ejemplo, nosotros tenemos una moto grande e íbamos a todos lados con moto, a ella le encanta ir en moto y tal, pues llevamos cerca de… este verano apenas hemos cogido la moto, bueno, yo la he cogido para ir a nadar, he vuelto y cuatro cosillas. Pero lo que es ir los dos juntos, que nos gustaba ir a tal sitio y tal pues no… apenas la hemos cogido.

**7:27 Y ¿ve que ha afectado un poco, desde el diagnóstico, a la relación de pareja?**

Bueno, sí, hombre, la relación de pareja no afecta tanto porque, bueno, yo no sabría qué hacer sin ella, la verdad. Entonces yo aguanto lo que haga falta, aunque cuesta, cuesta trabajo. Pero bueno, pues eso, tirar para adelante, ¿no?

Entonces, hombre, es más duro, ya no podemos viajar como viajábamos antes, se cansa enseguida, y tal ¿no? Entonces… sí, se puede decir que sí, que es duro, sí.

Sí porque miras a otras personas, miras a otras parejas y dices, ojalá pudiéramos hacer cosas tan sencillas como darse un paseo en bicicleta los dos, no? Por ejemplo.

Me acuerdo que montamos la bicicleta, todo el sistema este, yo compré un sistema para las bicicletas, te tiras 20 minutos para montar el tema y ella, a los 10 min de estar en la bicicleta ya se tuvo que parar. Entonces ahí hubo una discusión, porque le dije yo, “Joe, para 10 minutos…” ¿sabes? Para 10 minutos, montar todo esto y para arriba y para abajo… Ahí no fui yo muy…

Ese tipo de cosas ocurren mucho, ¿no?

**8:30 Claro desarrollar un poco la empatía se va haciendo poco a poco también, ¿no?**

Poco a poco, sí. Pero yo veo que cometo fallos en ese sentido muchas veces. Incomprensión. De no entender, ¿no?

“¡Ah! ¡10 minutos y ya está cansada!”

Y dices tú: “¡Joe, está cansada! Pero, ¡Coño! Es que… ¡No me ha dado tiempo a calentar y ya tenemos que dejar la bicicleta!”

Entonces después ves otras parejas que, por ejemplo, ellos cogen sus bicicletas, o van andando y, o hacen senderismo, por el campo, que relaja bastante y nosotros no, no podemos hacer eso, ¿no? Y eso pues es aguantar y ya está a ver si la cosa se va mejorando.

Ya te digo, con el medicamento ese ella se empieza a sentir un poco mejor.

**9:10 ¡Qué bien!**

**Y a nivel de deseo, porque, por ejemplo, hay muchas pacientes que refieren que con la medicación, que se sienten como más hinchadas…**

Deseo, ¿a nivel sexual, dices tú?

**Sí, eh…**

Sí, tiene menos, sí.

**Se nota, ¿no? Y, ¿cuándo se toma más medicación lo nota también?**

Sí, también se nota. Por ejemplo, lo podemos hacer una vez a la semana. Últimamente, a lo último ya es, una vez cada dos semanas, por ejemplo, cada tres semanas, inclusive. Hemos tenido bajones de una vez cada tres semanas.

**9:50 Porque ella siente dolor, ¿no?**

Ella sobre todo, lo que yo veo, es que no tiene ganas. Por ejemplo, ten en cuenta de que las posturas, pues apenas se pueden practicar posturas. Apenas podemos hacer ningún tipo de postura… yo encima y, poco más, ¿me entiendes? Para que ella no sufra nada. Entonces eso también limita bastante la vida sexual, lo que es la apetencia de sexo. Ten en cuenta que si siempre lo haces de una misma manera, bueno, pues se hace, pero cada vez menos. Y sobre todo por parte de ella el tema de los dolores y eso. Yo lo que veo es que tiene menos ganas, sí.

**10:27 Y por ejemplo, ¿el deseo por su parte hacia ella?**

Baja un poco también, claro. Ten en cuenta que al no hacer las cosas que… al no practicar el sexo en toda su plenitud, como se hacía antes, pues eso hace también que se baje. Es lo mismo, es hacerlo siempre de la misma forma, arriba, monótona y tal, pues también hace que se acorte un poco el deseo, sí.

**10:54 Y por ejemplo, a nivel de información, ¿qué alternativas encuentran al coito?** **Todos estamos acostumbrados a que nuestra sociedad es muy coitocentrista, y es como: “esto es hacer el amor y otras cosas no”. ¿Han buscado alternativas?**

No lo hemos hecho. La verdad es que no, porque ya te digo que ella ya, se ha reducido, se ha reducido, yo creo que por parte de los dos, también en parte. Pero yo creo que a ella le ha propiciado más. Entonces no, no hemos buscado… simplemente, bueno, estamos bien juntos, muy bien, la verdad, y salimos, a lo mejor, vamos a bares, a restaurantes, también, bueno también por la noche, vemos mucho la tele los sábados por la noche, que digo yo alguna vez: “esto es un poco…” [risas] Pero nos gusta, la verdad es que nos gusta, ¿sabes? Nos ponemos allí nuestras cositas y nos hemos adaptado a eso, pero el tema del sexo lo estamos dejando de lado, sí, está quedando de lado.

**11:54 Y por ejemplo, ¿pensarían en algún momento el hablarlo y el poder ir a algún sitio para conocer un poco más de información acerca qué posibilidades hay…?**

Claro, tarde o temprano, como esto siga así, tendremos que ver algo de eso. Sobre todo yo, lo que estoy ahora es esperando, también, a ver si va, si mejora un poco y eso, para hacer algo sobre el tema ese. Pero tarde o temprano lo tendremos que hablar. Es una parte muy importante de la pareja, y está como muriendo un poco. Lo que pasa es que bueno, yo no creo que eso acabe con la pareja, sinceramente. Aunque el sexo es muy importante para la pareja y tal pero, tal y como somos los dos, no creo que seamos… Yo no creo que me vaya a ir, sinceramente, con otra persona porque no tengo suficiente sexo con ella…

**12:41 Hay cosas más… Más fuertes, ¿no? En la pareja….**

Y bueno ya, ya tengo 46 años y la verdad es que, en mí, no sé qué ocurre, pero… no sé por qué será, pero no tengo una actividad sexual… no estoy yo como muy toro, vamos [risas] Lo estaba antes pero ahora ya como que no… que le doy importancia a otras cosas, no me parece muy, muy importante, entonces, puedo aguantarme, digamos. Tampoco lo deseo con otras personas, realmente, vamos.

**13:11 Son etapas de la vida, en realidad, uno no está siempre como a los 19 años, ¿no?**

Exactamente. Después, a lo mejor, a los 30 años sí hemos tenido etapas que hemos estado muy fuertes sexualmente, hemos hecho cositas y tal. Pero ahora mismo la verdad, es que yo por lo menos, no sé, por el tema de mis padres, que están chungos también… me rodea mucho la enfermedad ahora mismo a mi alrededor. Entonces la verdad es que eso me tiene un poco… Y también he cambiado un poco. Nosotros cambiamos de entorno laboral, ahora estaba allí , ahora estoy aquí, es diferente, compañeros, la casa, que has cambiado de vida, antes estaba solo, ahora estoy todo el rato conviviendo entonces como que… he aparcado… yo he aparcado también un poco el sexo…

**13:53 A nivel personal incluso, ¿no?**

Hay poca demanda por parte de ella también, entiendo que la haya, ¿vale? Y ¿qué voy a hacer? Tampoco… no me voy a amargar por eso ni mucho menos.

**14:06 Claro…**

Y es que yo, ya te digo, tengo… tengo otras cosas también

**14:12 [risas] Hay muchas cosas en las que pensar, claro.**

Sí, yo es que no sé. Mira que cuando yo era joven era, eso, pues como todos los jóvenes… pero ahora como que le voy dando menos importancia al sexo, no sé. Supongo que no será bueno, pero es la verdad.

**14:27 Es tan bueno como malo, es… si uno está cómodo y uno está bien, todo está permitido.**

La verdad es que sí. Y yo no cambiaba a mi mujer por ninguna mujer del mundo. Y si me faltara pues me moría, la verdad. O sea que…

**14:42 Lo ideal de la sexualidad es vivirla como uno quiere, es decir, que si alguien quiere ser gay, que sea gay, que si alguien quiere tener mucho deseo, que tenga mucho deseo, que si alguien quiere tener poco, que tenga poco… si nunca hace nada que moleste al otro, y siempre están los dos de acuerdo, uno puede estar en cualquiera de los límites de lo que quiera…**

Si yo me adapto. Mi forma de ser es que yo me adaptaría. Cuando la cosa esté mejor, porque nosotros no perdemos la esperanza de que la cosa vaya a mejor, que haya más demanda, yo creo que habrá… habrá un rebrote, ¿no? Ahora mismo, al no haber demando yo no soy tampoco… como que me vengo abajo un poco. Y no le voy a echar en cara a ella, ni nada de eso, ¿no?

**15:29 Y, ¿qué recomendaría a una persona que su pareja acaba de ser diagnosticada de Fibromialgia?**

Pues hombre, yo creo que tienes que querer mucho a tu pareja, porque es duro. ¿Qué le diría? Pues que… que es muy difícil. Tratar de sensibilizarse, que yo creo que es lo más importante. Porque en mi caso concreto, ya te digo, algunas veces es difícil, porque ella parece que está siempre bien, aunque no lo esté, pero da esa…

**Positiva, ¿no?**

Sí, es muy positiva. Muy, muy, muy positiva.

**Qué bien.**

Yo soy un poco más negativo y ella es muy positiva. Es una de las cosas que más me gusta de ella. Lo positiva que es.

**Qué importante.**

Sí, yo creo que sí, que es fundamental. Sobre todo viniendo de mi, que yo no soy tan positivo. Entonces lo valoro.

Y bueno pues que, que tenga paciencia, y sobre todo que sea muy sensible hacia esa persona. Y bueno, que si la quiere pues que… bajar las expectativas de lo que quieres hacer con ella. Y bueno, y si, si no podemos ir a Edimburgo, a viajar durante una semana pues vamos a Marbella a un hotelito, por ejemplo.

**Claro.**

Y ya está.

**Y uno se ahorra el avión, que es muy pesado, también… [risas]**

Y bueno también es importante el rodearse de amigos…

**17:01 No dejar nada sin cubrir…**

Claro, tener entorno social también, ¿no? Y bueno, y también pues respaldarte con hobbies que tengas y eso, sobre todo para llevar tu el… no meterte tanto, ¿no? Sino tener un poquito tu también y relajarte, ¿no? Y ya está. Eso es lo que yo recomendaría

**17:22 Qué bien.**

No tomárselo en serio

**No tomárselo en serio…**

Yo creo que la vida no te la tienes que tomar muy en serio. Cuando te viene una cosa así pues, tirar para adelante y ya está que hay momentos duros también, que hay momentos en los que dices, momentos en los que eres egoísta y dices: “No sé si a lo mejor, estaría mejor solo, porque podría hacer…” Pero después lo piensas y dices: “¡pues no!”. Yo lo he pensado eso. Me he planteado esa situación. Y luego a lo mejor es que a mí tampoco me viene bien, que a lo mejor, yo aquí me estoy hundiendo porque yo no estoy mal, y tal. Pero después dices: “¡Yo no estaría mejor sólo!” Yo creo que no, que no estaría mejor solo. Prefiero acarrear y estar, estar con ella, y estar… estar ahí sentados en el banco tomando el sol y estar con ella… Sí. Sí.

Me he planteado ya el vivir solo, que yo puedo ser muy deportista, y podría hacer muchas cosas, y dices: “No, no estaría mejor.” Sinceramente no estaría mejor. Si lo que puedo hacer no lo puedo hacer con ella, no…

**18:32 Claro… Qué bien. Muchas gracias.**

Cuando he estado hablando con su mujer me ha dicho que llevaba unos seis meses de baja.

**¿Hace cuánto tiempo le diagnosticaron?**

Pues eso fue a mediados de junio (fecha actual febrero 2017), pues se sentía mal, tuvo que en el trabajo no se sentía bien. Llevaba ya tiempo con dolores, lo que pasa es que es una mujer que aguanta. Y, de hecho, un mes antes de darse de baja, estaba yendo al trabajo pero no cogía el coche, lo mismo la llevaba yo al trabajo que una compañera la traía y demás. Y resulta que es que no podía, no podía con el coche. Ella llegaba allí, empezaba la hora laboral y ni aguantaba. Pero era una mujer… es de mucho aguante. Y en vista de que no, que una vez ya no podía más, tuve que ir a recurrir a ella allí al trabajo para llevarla a urgencias y la vieron allí y la trataron porque estaba tomando pastilla y le sentó mal y vomitando y demás. Y ya a partir de ahí empieza ya el engranaje ese de tener que ir de un médico a otro, hasta de pedir cita ya para el traumatólogo que es único que le puede ver, de hecho Nicolás (médico de familia) le dijo: “venga para el traumatólogo, pide cita.” Y eso fue ya a finales de julio. Y ya la vio el traumatólogo, y de entrada ya le dijo:

-“¿Qué le pasa a usted?”

- Pues tengo dolores aquí…

Y ya, no dijo el diagnóstico pero, creo que ya tengo una leve intuición de que eso es… Y dijo la palabra: FIBROMIALGIA. Y entonces ya, le dio la baja, y a partir de mediados de julio hasta la presente, sigue de baja. Baja laboral.

**2:02 ¿Y se encuentra mejor? ¿Toma medicación?**

Toma medicamentos pero no le están surtiendo el efecto deseado. Porque eso es como te digo, ella tiene el dolor latente, por todo el cuerpo, latente, y ella, entre comillas, está haciendo vida normal con ese dolor. Pero cuando llega una crisis, un episodio de dolor fuerte, no puede, no puede aguantar, tiene que estar echada en el sofá, y al rato no puede estar en el sofá, tiene que estar andando, un dolor generalizado por todo el cuerpo. Como si tuviera en la espalda, de forma descriptiva, como una mochila llena de piedras. La espalda fuerte. A veces dolores localizados en cualquier parte del cuerpo, que de hecho se ven hasta bultitos. Dolores que los tiene localizados tienen bultitos. Y dice: “¡Mira, mira, toca!” Y yo le toco. “¡Pues es verdad!” Y ahí le duele. Y ya te digo, el dolor lo tiene, lo que pasa es que ella está conviviendo con él. Ya de tanto tiempo, lo tiene… pero cuando llega el episodio, que eso en la semana puede ocurrir un par de veces, son episodios fuertes, generalmente en tiempo así de frío, lluvia, humedad y demás… fuerte, fuerte. Y no puede aguantar, no puede aguantar. Hace la vida normal, entre comillas, lo que es en casa, las cosas de casa, lo que es sus labores, por decirlo así, la comida, pero se tiene que sentar, o tiene que eso. Cosa que en el trabajo… es imposible de realizar. Y estamos en ello… y está ella… así, viviendo, conviviendo con ese dolor. Y nosotros, los que convivimos, mi hija pequeña de 13 años y yo, lo estamos viendo el día a día. Y yo puedo dar fe y constatar que es un dolor constante, un dolor real, y que para nada se puede decir que es…

**3:56 Inventado…**

Inventado o, como se dice en términos… psicosomatizado, o demás… Para nada. Ella cuando le dice: “Bueno tu tu tranquila, tu ve viendo, tu tomatelo con calma, no tengas…(como si tuviera depresión) “

Y ella de depresión nada. Ella dice: “Yo no tengo depresión, yo lo que tengo son dolores.”

**4:22 Claro…**

Y cuando uno tiene unos dolores fuertes, intensos y muy agudos, no lo puede soportar. No lo puede soportar. Y yo me tengo que hacer fuerte con ella, pero por dentro se queda, pues… como decimos aquí mucho, la pena dentro. De ver a esa mujer, tiene 51 años, 52, pero es una mujer muy activa, siempre ha sido activa. Positiva, activa. Y gracias a esa positividad que tiene, sigue manteniéndose en eso. Pero cuando la veo yo de esa manera, ahí se pierde ella, se pierde la pobre. Y yo tengo que hacerme el fuerte, no por mí, ya por mi hija que tiene 13 años que no lo viva tampoco como es. Pero, sinceramente, es una enfermedad extraña, muy rara, y a ver si de verdad, podemos hacer algo por…

**5:15 Es desconocida, entonces quizás, conociéndola un poco más, se puede entender. No entendemos lo que no conocemos.**

**Y, por ejemplo, a nivel de tareas del hogar, ¿cómo lo reparten?**

Las tareas del hogar, hace lo que puede, ya a veces, como mi colegio, yo trabajo en el colegio, ¿no? Soy maestro. Pues nos quedamos algunos días en el colegio a medio días porque mi hija da una asignatura para el Cambridge y demás, pues nos quedamos allí para comer, y no vamos a casa. Lo hemos hecho de esa forma para que no tenga una sobrecarga de tener que hacer la comida ni nada. Pero los días normales, hace lo que puede. Hace lo que puede. Y yo lo que pueda ayudar, yo siempre ayudo. Lo que pasa es eso, cuando ella antes trabajaba, estando normal, entonces yo hacía las labores, eso no es ayudar, es una cosa de los dos.

**6:11 De equipo.**

De equipo, exactamente. Eso no es ayudar, eso es: tú estás trabajando un sábado, o un lunes y yo no, pues yo hago lo que tenga que hacer.

**6:18 Claro.**

No hay problema. Eso no hay. Pero claro, ahora mi trabajo es todo el día y cuando llegamos a medio día, pues, lo que haya, sino pues cualquier cosa que se pueda improvisar y yo le quito los trabajos pesados, duros, de coger… o cosas que…, o de ropa, o de la canasta que está ya llena, o el lavavajillas cuando cojo los platos… yo lo hago, yo, por tal de que no se esfuerce, no haga ella un sobresfuerzo que le pueda dañar o algo. También es verdad que hay que dejarle autonomía, para ella valerse por sí misma, tampoco… eso le puede también…

**6:58 Eso puede ser contraproducente, ¿no?**

Exactamente. Y uno hace lo que puede. Pero la verdad es que es muy dura. Es muy dura esa enfermedad.

**7:07 Y por ejemplo a nivel de comprensión, ¿ella piensa que usted la comprende y que comprende la enfermedad?**

Sí, ella ya, ella lo entiende. Ella la entiende. Ella cuando está así yo… uff, pues… uno… está… es normal. Uno habla, pues saca el carácter y es normal, pero yo, puedo asegurarte que la comprendemos y estamos para lo que sea.

**7:33 Una de las cosas de las que se quejan mucho las pacientes es que dice que van al médico y el médico no las comprende, en casa nadie las comprende, en el trabajo nadie las comprende, y que todo el mundo les dice que se quejan, se quejan, y ya está. Entonces, yo creo que por lo que me he leído hasta ahora y por lo que he podido conocer a nivel personal, la comprensión por parte de los demás, hace que la autoestima de la paciente esté mucho más….**

Sí, por supuesto. De hecho, el médico de familia, Nicolás, por supuesto, un hombre abierto, y sabe lo que tiene ella, porque nos conoce desde hace tiempo, mucho tiempo, y ella es raro que se dé de baja por gusto. Y la ha estado tratando, y eso lo ve ella. Él la conoce de primera mano y sabe cómo es ella, su personalidad, y cuando la ve ahí sentada, de esa manera… “¡Uh, tú no estás bien!” Y de hecho, la primera vez que, de las primeras consultas que él estuvo viéndola, ella no podía… estaba rígida. Rígida, no podía… Y él lo notaba, se daba cuenta. Poco a poco, con una cosa, con otra…

También es bueno que está haciendo ella, caminatas, andar. Yo me sumo a ella, por estar con ella, porque, si uno es egoísta, dice: “bueno, a mí me gusta andar, mis caminatas, mis paseítos, yo me pongo mis casquitos, mi musiquita…”

Pero esto no es un sacrificio, por decir, bueno yo me quito y me voy con ella, al contrario, yo le digo: “mira vamos a dar una caminata.”

Y damos una vuelta de una hora o por ahí, andando, esto y lo otro. Para que estemos los dos charlando. Porque eso es bueno, para que haya entre nosotros una… y hablamos, y hablamos y demás. Y eso viene bien, lo que pasa es que algunas veces vienen más, como decirte, más reventada por el tema de la… (Fibromialgia), pero le viene bien. Y uno hace lo que pueda, lo que pueda. Si por mi fuera, pues estaría… eso es, lo que tienen dentro ella…

**9:32 Y, por ejemplo, la medicación muchas veces hace que, según los casos, las pacientes se hinchen un poco y dicen que se sienten menos atractivas. ¿Es el caso de su mujer?**

No ella, ella pues… lo que le pasa es que hay un hándicap que es el trabajo, porque si sale fuera, y a ella le apetece salir fuera porque, cuando pueda, siempre cuando pueda, siempre y que conste en eso, con el dolor latente…

**10:03 Eso es constante…**

Ella no lo va a decir siempre… me duele aquí, me duele aquí… porque es donde más le duele, ¿no? Tiene que salir.

**10:11 Claro…**

Que si se queda mustia en un rincón ahí, no le va a venir bien tampoco. Pero de hecho, si, estamos hablando de tema laboral, si alguien la ve, que es de otro sitio, ya puede entender lo que pasa… que dicen: “mira, y está de baja, y está con el marido”, por ejemplo, ¿no? Que ya sabemos que hay mucha suspicacia en eso, ¿no? Pero que no. Y ella tiene el ánimo de eso. Otras veces no lo tiene. Cuando está, ya te digo, cuando está con el dolor intenso, es que ni puede salir a penas. Cuando no pues… hay que darle a estas personas actividad, movimiento… no dejarlas ahí aparcadas y ya está. Y siempre con la pena, con la pena. Hay que sobreponerse, siempre, siempre, aunque lo tengas, aunque esté así. Y ella de su parte lo pone. Pero ya te digo, son dos caras, una la que está medio bien, y otra cuando no está, ya no es “mi Ana”. Vamos a decirlo así. YA ES UNA MUJER QUE NO PUEDE CON SU ALMA.

**11:11 Un cuerpo que duele…**

Un cuerpo que duele por todos lados. El otro día se le quedó engarrotada una mano, por estar tres minutos con el móvil. Y después: “¿Qué te pasa?” – “¡Que no puedo, no puedo!” Con agua caliente, un rato. La mano ya encendida. Enrojecida por completo, hasta que poco a poco ha ido articulando. Pero, no sé. Los tobillos los tiene hinchados, las rodillas, yo creo que hasta deformación del cuerpo, se le está notando ya algo.

**11.40 Pero, ¿a partir de la medicación o antes incluso?**

Uf…(suspiro pensativo) Yo te digo ahora. Ella está tomando sus medicamentos. Pero también está tomando cosas de herboristería y demás que siempre el colágeno y demás. Cosas que le pueden ayudar para el tema de los huesos y los músculos. Pero yo la veo que está… no engordando, pero las piernas ya las tiene algo más deformadas, las rodillas, los tobillos los tiene como hinchados…

**12:08 Bueno, pero si va andando y eso… eso es lo**

Sí, que vaya andando… eso es lo bueno. Ahora lo ha dejado porque como el tiempo no acompaña… pero ya le digo yo: “tu, aunque no esté yo, tú ponte a andar.” Es una mujer decidida, es una mujer que ha luchado mucho por su vida y las cosas. Yo la veo muy valiente, muy valiente. Y si tienes tú una persona valiente, tú tienes que ser más valiente.

**12:31 Claro…**

Tú no puedes ahora…

**¡Achicarte!**

¡Exactamente! Tú tienes que venga, pues venga vamos para acá. Y con la niña, que nos reactiva más todavía. Que somos ya mayorcitos, ¿no? Pero la niña con 13 años, ¡pues venga! Y eso es un plus más que te hace darte más energía.

**12:48 Claro…**

Y hacemos lo que podemos y hacemos la vida normal familiar, entre comillas siempre. De una forma relativa. Pero se hace lo que se puede.

**12:56 Y a nivel de deseo sexual, por ejemplo, ¿cómo ha influido la medicación o la enfermedad en ella? Quizás ahora tiene menos ganillas, o…**

Bueno, eso, nos lo podemos reservar para nosotros…

**13:11 Vale, no, no…**

Eso es una cosa que…

**Yo… son las preguntas y si no quiere contestar**

Te entiendo, no es no querer contestar pero es que es una cosa más personal y…

**Por supuesto. Y no quiero que le siente mal.**

No, por favor, por favor, para nada.

**Es uno de los temas que se dice que con la medicación influye…**

Sí, sí.

**Entonces, queríamos… porque muchas veces, las pacientes dicen que si toman la medicación se sienten menos animadas, para hacer cosas en general, social, y a nivel de pareja también. Vamos, que es una pregunta que, por los estudios que tal…**

Lo entiendo.

**Pero que no tiene que responderla.**

Por mi parte es algo muy personal, si le preguntas a ella, pues ella te podrá responder o te podrá lo que sea… [Risas]

**13: 55 ¡Estupendo! ¡Estupendo!**

**Y, por ejemplo, una pregunta que es lo que, más o menos, para esto es el estudio… a una persona que su pareja tiene Fibromialgia, se lo acaban de decir, cómo pareja, ¿usted qué le recomendaría? ¿Qué diría? Pues que no se olvide de… de ser muy comprensivo, o de tal o de cual… ¿cuál es la lista de cosas que usted le podría recomendar a alguien? ¿a otro hombre en la misma situación?**

Hombre, lo primero está el shock que llevan los dos y el que se lleva la persona en sí. Pero por la poca experiencia que estamos teniendo le podría decir que: ¡¡ánimo!! Que es una enfermedad rara, una enfermedad extraña que parece que no tiene curación pero podemos convivir con ella, que puede eso, haciendo un, tratándole al cuerpo de una manera o de otra, que ánimo porque puede hacer gimnasia, puede andar… te comentó que hacía Chi Kung, que está haciendo cosas, ella por su cuenta ha buscado formas y maneras. La filosofía del Chi Kung es: al amanecer tiene que estar en lo algo de un monte haciéndola… Yo iba con ella a acompañarla y ella se quedaba con el maestro y las demás personas, yo no, yo me quedaba esperando o me daba mi caminata por mi parte, pero a esa hora. Todos los días.

**15:23 ¿A qué hora?**

Depende, en verano los viernes, sábados y domingos, porque es cuando podíamos, a las 7 de la mañana a hacer Chi Kung.

[Risas]

**15:33 ¡Qué bien!**

¡En una colina! [Risas]

Y claro, ya con el tiempo, que tampoco ni acompaña ni nada (es febrero), pero yo la acompaño, hombre, al principio uno recién levantado… mi verano, que es mis vacaciones, uno que es maestro, que tienen las vacaciones como los niños, dices, bueno. Pero yo lo hacía por ella, porque ella no podía coger el coche, tenía que desplazarse a Fuengirola a hacer eso. También a hacerse masajes, era el hombre, también es fisioterapeuta pero de cosas orientales de Tsiatsu y demás. Yo estaba con ella ahí y apoyándola en lo que sea. Donde ha ido ella la he acompañado yo. Y donde vaya ella, también.

**16:15 Eso es muy importante. Qué bien.**

Pero también hay que decir que ahora se le puede dar un poco de autonomía, a lo que pueda ir ella que vaya ella también. Que sino creas una dependencia… que si no estoy yo que soy el bastón, entonces no sabe sostenerse, en plan así simbólico, entonces yo lo que quiero es que ella… ella puede. Es una mujer que ha luchado mucho, una mujer fuerte, de… más fuerte que yo si te digo. Porque a mí si me pasara eso, no sé cómo sabría reaccionar en ese aspecto. Pero bueno, siempre te pone la vida a prueba y esas pruebas que te hace tu mismo te sorprendes de cómo la has sacado adelante…

**16:53 Es verdad, ¿eh?**

Sales adelante como el ave fénix, resurges de las cenizas y ya está, y de otra enfermedades también, que las hay más graves, y eso a la persona que es valiente tiene que luchar, y querer es poder y eso nuestro lema, que querer es poder y que siga así. No sé los años cómo serán, porque esta enfermedad es tan rara que no sé si va a haber deterioro al cabo de los años, pero ahí estaremos…

**17:22 No se conoce exactamente por lo mismo, porque hace pocos años que se empezó a diagnosticar, entonces no se, como que no se ha seguido, a nivel longitudinal no se ha seguido ningún estudio que sea bueno, que sea consistente y que pueda arrojar luz tampoco.**

Claro.

**17:45 Pero lo que sí hemos visto en estudios anteriores que han hecho, por ejemplo, mis profesores allí en Almería, que están muy metidos en el tema, es lo importante que es la pareja y casi toda las mujeres, que se entrevistaron hace ya dos años, hablaban de lo mismo, de lo importantísimo que es el apoyo de la pareja. Y eso, que muchas veces, aunque la pregunta fuese, lo siento, un poco así, decían que eso era algo muy importante para ellas y que se sentían que hay que ver que quizás se esperaba más o lo que sea. Pero luego, por otro lado, por eso se quiere ver qué piensan también los hombres, se ve, los hombres, por otro lado tampoco, vamos, que están contento con que esa mujer esté luchando, esté tal, que todo es mucho más normal de lo horroroso que lo ponen, pero aunque la enfermedad sea difícil que hay como… que si el apoyo de la familia y de la pareja es fuerte, normalmente, va todo mejor, es una situación en la que al final, uno se termina adaptando.**

Sí.

**19:05 Quizás la persona que lo sufre, menos, porque los dolores están ahí, pero que el entorno, aunque al principio sea como un poco tsunami, que llega la ola ahí y nadie sabe cómo va a poder respirar y… que luego llega un momento en el que sí, que se crean, pues como con cualquier problema que aparece en la vida, se van creando unas tablas y uno va poniendo y va subiendo y va estando mejor.**

Claro.

**19:29 Y claro, y la familia y la pareja son pilares fundamentales.**

Desde luego es un factor muy importante.

**Porque luego no hay otro tipo de apoyo social. Porque, ¿a dónde va una persona? ¿a qué hombro llora? Si no tiene familia, no tiene pareja. Entonces eso es lo que se ha visto que es lo que más ayuda. Por ejemplo gente que no tiene pareja, o que no tiene familia, lo pasa bastante peor.**

19:55 Lo pasa ya mal si se encuentra solo o personas que no la comprenden y demás, pues sí.

**20:00 Y van al médico y no las entienden, van al trabajo y no las entienden…**

Puede afectar muchísimo también a nivel anímico y demás, claro, por supuesto.

**Claro. Y entonces se quiere ver eso también, el comparar un poco y decir: bueno, entonces estas personas, si no tienen pareja, ¿qué otras cosas podrían hacer para que el impacto de la enfermedad no sea tan grande?**

Claro, sí sí.

**20:22 Hay tanto que estudiar y tanto que ver.**

Sí, desde luego.

Estamos ahora empezando, esto es el embrión ahora, a ver si…

**20:29 Hay estudios y hay cosas…**

Hay estudios, por lo que he estado informándome, cosas divulgativas sobre el tema, en Estados Unidos y demás.

**Y aquí en España hay bastante.**

Sí, ¿no?

**20:41 Sí. Porque yo creo que, como es eso, este tipo de estudios se tiene que hacer a través de entrevistas, es como un estudio antropológico, de campo casi, se tiene que hablar, y para hablar muchas veces pues, quizás uno si se va a Suecia, la gente no habla tanto como aquí…**

Claro, normal.

**21:00 Claro, son culturas mucho más cerradas y eso, y entonces, aquí en España sí se están haciendo muchos estudios y hay gente que está sacando cosas. Luego también es eso, muchas veces se ve solo el paciente, pero ¿y la repercusión que tiene en su entorno? Es que ahí eso es importante. Que siempre nos olvidamos de los hijos, de los maridos, de los padres y de las madres, es que hay niñas que tienen 20 años.**

Es que es un efecto colateral, si se puede llamar así de esa forma. Es normal.

**21:29 Y eso es muy importante.**

Es que estamos para eso, cuando una persona está con otra… Yo siempre aplico frases muy localistas, estar para las verdes y las maduras

**21:40 [Risas] A mí me encanta también. Las frases hechas**

No lo voy a decir muy andaluz porque… yo soy muy malagueño, y no lo puedo evitar, pero yo aplico muchos términos y mucho argot de los que vivimos el día a día.

**21:53 Claro.**

Y hay que estar para las verdes y las maduras. Y si uno está ahí, hay un compromiso, aunque la palabra suena fatal, compromiso. Cuando son dos personas es una, y si dos personas que se unen forma una, y esa ese uno ya es el centro motriz de la familia y mi Ana y yo, o yo y mi Ana, somos uno. Lo que pasa es que claro, a veces tenemos que estar separados por el tema de mío laboral. Pero cuando estamos juntos yo siempre le digo: “Tu haz lo que tengas que hacer” Porque es una mujer que ha luchado mucho. Su historial y el mío, cada uno por separado, pero ella ha luchado mucho por sí misma. Es una mujer batalladora, luchadora, ¿se va a amendentrar ahora? Tú eres fuerte.

**22:50 ¡Qué buena actitud!**

No es que es así. Y ella es así. Quizás, a lo mejor, yo, yo he pasado también mis temas con mi gente y eso. Pero que ella ha estado ahí. Eso no es un pacto ni es nada, es que surge en el momento. Eso es como una flor cuando sale, ¿por qué sale la flor de día con la luz? Pues igual nosotros, nosotros nos iluminamos uno al otro y decimos: “bueno, esto es así”. Ahora tenemos, yo 54 y ella ya… cuando lleguemos a más mayorcitos, seguiremos estando, y todo lo demás es secundario. Y tenemos que estar. Ahora tenemos que seguir para adelante con nuestra hija la pequeña, porque tiene ella una mayor, que es médico,

**23:34 ¡Ah! ¡Qué bien, ¿no?! Porque, hombre, eso ayuda.**

Sí, tiene 31 años. La conocí con 7 años, es como mi hija. Y ella es médico y demás, pero ahora tenemos a esta, para no aburrirnos dijimos: “venga, vamos a buscar una más!” Y venga, para adelante. Y ya está. Y nuestra niña es, es nuestra reactivación.

**23:56 Claro.**

Porque sino estaríamos… pues, a lo mejor no habría un objetivo de decir, “pues venga pues vamos a movernos”, ella es la que nos mueve. Y estamos así. Y mientras estemos así, nada. La pena es cuando la vemos de esa manera, pues ya está, estamos así. Y se le deja, y cuanto menos le hables en esos momentos, mejor. Y se hace la vida y la actividad normal, hasta que se le pase eso. Yo te voy a decir sinceramente que esta enfermedad la pasó mi madre antes de morir ya llevaba tiempo, y se estaba diciendo, te digo de hace 20 años,. Murió en el 2004, murió de cáncer de útero. Y estaba rabiando, pero antes ya le había dicho algún médico por ahí: “Usted tiene Fibromialgia” y los dolores los tenía. Y se le inflamaban estas cosas.

**24:47 ¿Los mismos síntomas? Parecidos, ¿no?**

Y después ya ese dolor que tenía en la parte del vientre, del endometrio que le estaba ya eso ahí… yo viví un parte muy dura de mi vida con mi madre. Después mi padre se murió… fíjate tú, yo te cuento ya mi historia. Por eso dice uno: “uno lo que ha pasado ya, lo que venga ahora…”

Mi padre a menos de los tres meses se murió después de mi madre, para que tú veas. Y yo hijo único. Yo he tenido que luchar solo, y ¿quién estaba al lado mía para apoyarme? Pues mi mujer estaba siempre. Siempre ella.

Mi madre tenía esos dolores: me duele aquí, tengo una hinchazón aquí, cada día.

**25:35 La pobre… y antes era todavía más desconocido todavía…**

Ya dijeron eso de… y cuando lo dijo el médico ese de… el traumatólogo, el veintitantos de julio: “usted, no me equivoco, usted tiene Fibromialgia” Se nos vino el mundo encima.

**25:54 Porque ya lo había visto usted antes, ¿no?**

Tanto a ella, por supuesto, como a mí, porque yo ya conocía esa enfermedad. Y ella ya la sabía, y ya sabíamos lo que era, lo que pasa es que estábamos hablando ya de una mujer de setenta y tantos años, claro, ahí la Fibromialgia, cuando la edad es más avanzada, más se ensaña con ella porque el dolor es más fuerte.

**26:15 Claro.**

Y el aguante es menos. Y tienen fuu… yo me acuerdo con nolotil pinchándole en la barriga, en el pompis, ya en ninguna parte se podía pinchar. Así que de practicante he sido yo de verdad. Había partes en las que no se podía pinchar. Que rompía la aguja. Y yo: “¡Dios mío, dame valor, dame valor!”

**26:39 Oy, eso es durísimo.**

Y yo tenía que estar… y muchas cosas, entonces, esto que yo he vivido antes, pues yo digo, esto no va… Mi mujer está todavía enérgica, y como todavía hay tiempo, a ver si se consigue algo, poco a poco, y como es joven puede moverse, puede estar activa, antes de que llegue a ser una ancianita y no pueda ya valerse por sí misma.

**27:12 Ella es súper positiva. He estado hablando con ella y es… Yo creo que la actitud hace muchísimo, y más teniendo un apoyo como el suyo, y una hija… yo creo que eso es lo que hace que uno pueda adaptarse bien a una situación tan difícil.**

La mente está distraída también. Está ocupada en una cosa y en otra. La mente no la puedes dejar en blanco y pensar siempre, cargarse a sí misma en eso. Tiene que estar activa. Tiene que estar haciendo, cualquier cosa.

**27:42 Y teniendo un objetivo como es tener una hija y tener un matrimonio que disfrutar y que… eso es súper importante.**

Lo vemos de verdad, todo de color rosa, pero tiene su cosa: la familia, la niña lo otro… vamos encaminados en eso y creo que lo estamos haciendo medianamente bien. Pero bueno, ahí estamos, a ver si mi mujer… hay algo ya que le quite eso. Uno piensa: “¿ será de estas enfermedades que vienen y se curan o se van? O algo que sigue, sigue, sigue…” Yo me paro a pensar por mí mismo, porque no quiero hablar con ella tanto de eso, porque sino, no quiero que sea el tema principal, quiero que no sea esto siempre, que sea otras cosas, o vanales, o superfluas, cualquier cosa, o otros temas serios, que no deriven siempre en esto.

**28:38 Que ha terminado siendo el centro de la familia, ¿no?**

Claro, claro, claro. Cuando está ella así, sí. Ella tiene que salir, sale, y algunas veces: “¿Tú me ves así? Pues ahora mismo estoy sintiendo dolores por aquí…”

**28:53 Claro, eso es lo que pasa.**

Eso es.

**28:56 Es que es tan difícil de comprender, porque dices: “pero si estás ahí…” ¡Y el esfuerzo que estará haciendo esa persona para estar ahí!**

Y es que nada más verla a ella y mirarle a los ojos y verle… se le nota. “Hoy estás tú… bueno, pues nada, venga, pues vamos a esto y a lo otro.”

Y darle ánimos y darle vidilla,

**29:14 Y encima influye mucho en el sueño…**

Yo es que soy muy activo, yo… y a los niños los muevo… Y no me gusta eso de estar…

**29:21 Estanco, ¿no?**

Yo, me gusta moverme y a los niños darle vida y eso, porque son niños. Y los niños otra cosa que te digo, trabajar con niños te reactiva también, es verdad que termina uno ya… vamos, hasta…

**29:34 Te roban la energía, ¿verdad? [Risas]**

Bueno, pero la tienes más, y sigues, sigues, y por una cosa y por otra, tiene que ser así. A parte de los conocimientos que les das, a los niños les tienes que dar… son niños de 8 o 9 años… y a mí me gusta hablar, hablar es difícil a niños de esas edades, no entiendes, esto y lo otro, si fuera gente más mayor, serían más eso, pero bueno… entonces, de esa forma que soy, de mi forma de ser, lo aplico a Ana, Pues, ¡ya está!

**30: 07 Toda esa pedagogía está muy bien, porque por ejemplo, lo de que no quiere que sea totalmente dependiente de usted, eso es buenísimo.**

Claro.

**Hay gente que sobreproteje a la persona que está enferma. Y el darle autonomía hace que esa persona no… pero eso es pedagógico totalmente. Eso es algo, que un profesor ´si lo puede saber.**

Eso es algo innato, eso es algo que, por mucho que hayas estudiado de pedagogía, psicopedagogía, didáctica, es una cosa innata, innata. Yo he vivido mucho ya, y yo he visto mucho, y yo veo y siempre digo: está lo correcto y lo incorrecto, tenemos que aprender lo correcto, tenemos que aprender lo incorrecto, para saber que esto no es bueno, y tengo que ir a por esto. Porque si nos encaminamos en un solo camino, valga la redundancia, después si te sales de ese, ¿qué hago? ¿qué hago?

**31:00 Claro, no tienes herramientas.**

Pues a ella igual, tu no te preocupes, y venga…

**31:07 Autonomía**

Eso, autonomía hay que darle, eso autonomía, y ella de hecho, sabe, no es de las que: “Ay! Ay! Ay!” ¡Ella no!

**31:39 Ella es muy resuelta y muy positiva.**

Desde luego, por supuesto. Si lo has notado, pues sí, es verdad, A ver si tienes el placer de conocerla.

**Nos interesa mucho saber qué ven las parejas, ¿qué sienten? ¿Cómo viven la enfermedad? ¿Cómo es, de repente, un diagnóstico?, ¿cómo cae en casa? o los síntomas, ¿cómo se viven? ¿Cómo es un día a día?**

Pues depende del día, porque, como te habrá dicho, hay días que está mejor y otros días que está peor. Y bueno, yo a mí hay muchas veces que se me cae el mundo encima, lo que pasa que no le quiero decir nada a ella porque, tú la has visto, es un poco, que el ánimo se le viene abajo. Pero bueno, lo vamos sobrellevando como podemos, ahora este año me he ido a unas vacaciones largas, necesitaba desconectar un poco, este invierno, y la ayudo en todo lo que puedo en casa. Porque es trabajo que le quito de estar agachándose, sacar el lavavajillas, poner fregar, esto y lo otro, pero ella sigue haciendo sus cosas, pero yo intento quitarle todo lo que pueda. Pero claro, es que hay veces que es, mira que no puedo, me voy a quedar en la cama un rato o se echa toda la tarde en la cama o ha habido mañanas que no ha podido incluso levantarse. Se ha levantado, ha desayunado, se ha acostado, así que bueno, intentamos llevarlo lo mejor posible. Es que otra cosa no se puede hacer. Es así.

**1:19 Claro. Y un día especial, por ejemplo, ¿cómo puede ser un día de una boda, un bautizo, una cosa así?**

Pues hay veces que tienes más ganas, más ánimo, menos ánimo, pero bueno, hay que ir, pues hay que ir. O sea que ella hace de tripas corazón ya va. Lo que pasa es que sí, pero por ejemplo, mira: el sábado pasado, estuvimos con mis tíos que nos invitaron a comer, nos dimos un paseo, fuimos a comer por encima de Velez-Málaga, nos fuimos a Torre del Mar a darnos un paseo, y bueno, salimos de la cafetería donde estábamos tomando café por la tarde, vamos, ya para venirnos, fui al servicio, ella se estaba fumando un cigarro, y ¡puuuf, estoy frita por llegar a casa! O sea, está pasándoselo bien pero a la vez estaba ya muy cansada. Y entonces estaba frita por llegar a casa. Yo también tenía ganas de llegar a casa, por quitarte la ropa o descansar, pero ella no. Es que dice: “estoy bien, que no es que se me esté haciendo pesado el día, pero estoy frita ya por llegar a casa.” Claro, llegamos a las 8 de la tarde desde las 11 de la mañana que salimos, las once menos cuarto… Que si va en el coche, con el paseo que dimos por Torre del Mar, se lo pasa bien, pero le cuesta la vida muchas veces. Es lo que yo le digo, lo que no te puedes quedar es encerrada en casa todo el día porque entonces es cuando no vas a salir. Ahora con este tiempo (febrero) pues a lo mejor te tiras 3 o 4 días, ahora vamos a dar una vuelta, vamos al Miramar (centro comercial), vamos a ir aquí, vamos a ir allá… Por tal de salir. Mañana, por ejemplo, vamos con mis padres, y vamos a tirar para Ronda.

**02:47 ¡Qué bien!**

Dice que no había visto Setenil de la Bodega, pues nos vamos allí a comer y después nos vamos a Ronda a dar una vuelta. Mañana llegará hecha polvo también.

**02:54 Claro.**

Claro, si mantiene más o menos como hoy después del masaje, pero ella sabe que tiene que salir también y hacer cosas. Porque tú no puedes romper la vida… que yo sé que le cuesta la vida. Hay veces que, ella por ejemplo, con las amigas, el primer viernes de cada mes, pues salen, pues lleva todo el invierno sin salir.

**03:18 Claro.**

Es que no me apetece, y menos con el frío. Claro, el frío le afecta mucho. Y yo: “pero chiquilla, sal y te distraes una mijilla” Y ella: “si yo sé que me lo paso bien pero que…” “Chiquilla vete un rato!” Y bueno, a veces que achuchándole… pero lleva todo el invierno que no sale. Que no sale. No sale. Pero es que dice que viene muy cansada.

Claro, date cuenta que salen por la tarde, para cenar e irse a tomarse algo después. Entonces claro, está todo el día y cuando llega ya por la tarde-noche, ella está ya reventada.

**03:45 Claro es que es lógico.**

Y claro, arréglate, componte y sal. Pues ya es un sobre esfuerzo. Al día siguiente está ya… ¡vamos, muerta!

[Risas]

**03:52 ¡Ay! ¡Pobre!**

Está muerta, pero bueno. Pero yo intento animarla por tal de que salga que se ventile un poco, que salga con las amigas, que bueno… los rollos que tenéis vosotras… Y hay veces que achuchándole, sale. Pero que no tengo ganas que no… Pero hay que hacer ganas

“¿A que tu no me dices que me vaya?”

No, porque no salgo, pero como fuera de estos tíos que están todo el día… puf, vería el cielo abierto…

Pero bueno, bien, dentro delo que cabe, bien. Lo vamos llevando como podemos.

**4:20 Y a nivel, por ejemplo, de comunicación de pareja,por ejemplo, creo que quizás no es el caso, pero hay muchas pacientes que dicen que los maridos no las entienden, o que no comprenden los dolores y eso, en vuestro caso….**

Si, yo sí que la entiendo, lo que pasa es que yo veo todo lo que sale de la FM. Son pacientes que las ves bien de aspecto pero no entiende… pero claro, yo llego un momento en el que…

Cuando túte has sido al servicio le he hecho algo así (Un roce) así, cariñoso, así, así, dice: “Uish! Ah!” Y se le ha puesto rojo. Dice: “Es como si tuviera un hematoma ahora mismo”. Pero bueno, si yo nada más que te he dado... y, y te tienes que ir cohibiendo y, voy a optar por no tocarte. Porque, cualquier caricia, que antes era una caricia, ahora es dolor.

**05:03 Claro, es como si fuese un…**

Llega un momento en el que ya me da miedo tocarte o algo, porque no sabes si te va a hacer daño o no. Pero bueno.

**05:09 Y a nivel de pareja ¿Cómo ha afectado la enfermedad?**

Pues, la verdad es que mal. Pero también lo vamos sobrellevando. No puedo forzarla a hacer una cosa que no quiera… y anivel de cama, todo, deposturas, de todo…

- “Ponte arriba”
- “No, arriba no puedo, no.”

Cambia todo, todo, todo, todo. De ser una pareja que, bastante activa, a prácticamente a pasar a nada. Y prácticamente como si tuviera una muñeca hinchable, o sea, ella disfruta pero procura no moverse, porque no puede moverse, entonces la flexibilidad que tenía antes, la agilidad que tenía que daba lo mismo arriba que abajo, que de un lado que del otro, pues… pues eso se ha perdido… y te tienes que adaptar tu también. Vamos para viejos… ay qué vejez me espera!!! Ay! Qué vejez me espera! [Risas] Ella se ríe, pero bueno…

Para eso estamos también….es que… ¿Qué quieres que haga con ella? ¿La voy a tirar ya? Después de tanto años…

**06:10 Y, por ejemplo, alternativas… nuestra sociedad es muy coitocentrista, como se dice, en plan, “palabra rara”, pero, por ejemplo, hay muchas alternativas que se pueden utilizar a la hora de mantener relaciones de pareja, quizás no como pensamos que deben ser satisfactorias, pero pueden ser incluso más satisfactorias. ¿Qué alternativas han encontrado?**

Bueno, alternativas, bueno, lo que te he dicho, cambiar de postura, utilizamos juguetes, para que estimule un poco más, pero… poco más. Yo por mucha imaginación que tenga, llega un momento en el que se te acaba.

**Claro**

Porque, por ejemplo, cuando estuvimos de viaje en noviembre, no fuimos 18 días por ahí, estuvimos 8 días por ahí de viaje y 10 días en casa de mi hijo en Zaragoza. El último día, en Santillana del Mar, me cogí una habitación con jacuzzi dentro, ¡hala! Y yo sabía ya que esa noche ya caía algo. [Risas] y ella ya estaba: predispuesta.

**Claro.**

¿Por qué? Porque ella ya sabía que eso era para lo que era. Pero después, sobre la marcha, como antes, que podía salir, así sobre la marcha… pues no. Ahora ya prácticamente, es previsto. Es previsto todo.

**07:29 Claro.**

Y de ella sale pocas veces. Yo muchas veces le digo: “Bueno, cuando vamos a hablar del Gobierno?” [Risas]

Esta tarde, nos vamos abajo y echamos la tarde y tal. Y a lo mejor acepta. La mayoría acepta, pero tampoco se lo digo, ni mucho menos, todas las semanas. Ella ve, cuando ve que llevamos ya un mes, un mes y pico, que estoy yo ya que me subo por las paredes, pues ya, acepta. Disfruta y tiene su orgasmo y todo bien. Pero no es lo mismo que antes, o sea, la facilidad de una pareja joven, o normal, que sube y baja… todo eso se ha perdido. Eso se ha perdido.

Ya le dijo… ¿quién fue? No sé… O lo leyó. Dice me falta, no tengo apetito sexual ninguno. Y no sé quién le dijo, es que no me acuerdo quien fue. Hombre si no tienes pues mira, pues veis películas, veis algo… total, últimamente vemos un rato algunas peliculillas o así, un rato, por tal de que se estimule un poco. La última vez: “Ah! Quita eso que paso de eso.” Y realmente, estaba excitada, pero lo que pasa que como también tenía los dolores que llevaba ya un tiempo, la penetración, imposible. Ya le pasó eso hace un año y pico, le mandó unos óvulos y le fueron bien y tal. Esta vez vinimos a ver si los óvulos aparecían por ahí, y no, bueno, entonces dijo Nicolás, bueno te voy a mandar una crema tal, bueno, de momento sigue. Hasta la cánula de la crema le molesta.

**08:56 Claro.**

Entonces, pues, lo que es penetrar, no hacemos, de momento.

**09:07 Y, por ejemplo, alternativas como masajes, como…**

Sí, eso sí, a ella le encanta que le dé masajes, eso sí. Le encanta. A mi también me gusta pero dice: “Ay! Tu sabes que es que me cansa mucho!” Vale, me gusta darlos, disfruto dándotelos, y sé que disfruta quien lo está recibiendo, bien. Pero es que a mí, de vez en cuando, también me gusta que me acaricien. Y antes sí, antes de vez en cuando, pero es que ahora: “es que tu sabes que no puedo, que no se qué no se cuanto” tampoco la puedes forzar a decir. Yo disfruto dándoselo, y ya está. [Risas] Pero vamos, entre col y col, caer una lechuga. Como dice el refrán. Y ya está.

**09:49 Y, por ejemplo, se sabe, bueno, ya lo ha dicho, que comprende la situación, que claro, que tampoco se puede forzar, pero ¿qué consejo le daría, por ejemplo, a una persona que, a la pareja de una persona que acaba de ser diagnosticada de FM?**

¿Con respecto al sexo?

**Sí, o de comprensión, sexual, de todo…**

Que lo intente llevar lo mejor que pueda porque eso va a ir para adelante. Eso no va a ir para atrás, o sea que… normalmente, esto son enfermedades que suelen ir…, bueno ojalá. Pero, yo que sé, que intente comprender a la otra persona, y que se tenga que adaptar a las circunstancia. A lo mejor hay mujeres que a lo mejor por la edad son, tienen cierta flexibilidad todavía, tienen, pero por los dolores que estoy viendo, me parece que da igual que sea joven o que vieja. O Sea, o más mayor.

**10:38 La edad es independiente en eso… Los síntomas…**

Por lo menos intentar comprender, ponerse en el pellejo de la otra persona aunque a veces te cueste mucho trabajo. Hay veces que te cuesta mucho trabajo, tú dices: ¡te veo bien y tal! Pero claro, es lo que sale en todos lados, dice: la ves bien pero después por dentro… Una simple caricia, es dolor. Es dolor. Una simple caricia es dolor… entonces, pues… dices: ¿coño! ¡Se te quitan las ganas de de tocar! Porque ya no sabes si le va a hacer daño, si no le va a hacer daño… y… ¡ya está!

**11:10 Y ha tenido en algún momento la idea o la necesidad de decir: “pues vamos a consultar con un especialista a ver si esto…”**

¿Un especialista de…? ¿De pareja?

**Sí, a nivel de pareja**

No, pensamos que no lo necesitamos.

**Claro.**

Hasta hace poco éramos una pareja bastante activa y… bueno ahora por circunstancias, son circunstancias. Que no es decir que ella no quiera. Que ella dice si… yo muchas veces, dice, yo no me, no me, no me da ganas, no tengo ganas. Bueno, si no tengo ganas, ¿qué hago? ¿la violo?

**No hombre [Risas]**

Me aguanto. Tu vas, te masturbas de vez en cuando y punto. Y digo: bueno, tiene guasa que a los cincuenta y tantos tenga uno que ir como si fuera un chaval de quince años, pero bueno… [Risas]

Por lo menos no te da otros pensamientos! Peor es el que se va de putas! Por lo menos yo me quedo en la casa!

**12:09 Sí, pero que hay… también, ya a nivel, no de la entrevista, pero que, muchas veces la gente piensa que eso, que el sexo es solamente lo que se supone que se puede… relaciones con penetración y demás…**

No hombre, ya sabes tú, que yo ya tengo asumido que si le duele, pues si no hay penetración, no hay penetración…

**Claro.**

Las dos últimas veces que le molestaba tanto, que la penetración imposible, la penetración no es posible, pues no es posible… pues nada, a tocar, y ya está!

**12:40 Claro, que habiendo más alternativas…**

Y que si me tengo que “reir” más con el clítoris que con la vagina, pues el clítoris, pues con el clítoris, ya está [Risas]

**Eso es otro mundo… Ahí se puede investigar…**

Si os da “gustirrini” a todas, qué quieres que te diga!

**Hay también muchas cosas que investigar también de si, por ejemplo, eh… Hay cosas que funcionan y cosas que quizás funcionan menos, pues hacer como un… una lista de cosas que van bien, y una lista de cosas que no…**

Yo sé que los juguetitos y el clítoris le funcionan muy bien, pues entonces pues ya está, perfecto… perfecto… De vez en cuando tengo que comprar un juguetito nuevo pero vamos, vas bien! El de antes, el otro… del otro te vas a aburrir ya mismo, como sigas así… [Risas] Pero bueno, funcionan, funcionan, y ella cuando se corre, se corre con ganas.

**13:22 Claro.**

Pero claro, si digo, coño! Si llevas un mes sin correrte es normal que te corras con ganas. Y digo es que… Si fuera todos los días pues a lo mejor no tanto, le pasa a uno, no es lo mismo estar todos los días que cuando estás una semana sin hacer nada, igual.

**13:33 Claro, y por ejemplo, después de tener relaciones sexuales con orgasmos, ¿ella se encuentra mejor? ¿Se encuentra más…?**

Sí.

**¿Al día siguiente está más, está mejor? ¿O estás más dolorida?**

Sí. Al otro día, La última vez, hace un par de semanas me parece que fue. Digo: ¿qué cómo has dormido?” Y me dice: “Bien”. ¿Relajadita, tranquilita, no? Te voy a tener que relajar todas las noches… Se echa a reir. [Risas] Pero sé que está mejor, como más relajada, o más yo que sé, si es que funciona mejor. Si tienes mejor cara. Se nota la que no hace nada y la que hace.

**14:05 La mejor medicina… [Risas]**

La lozanía en la cara se ve! Y sí, a lo mejor dice que ha dormido mejor, más relajada y tal, pero…

**Y a nivel de pareja también hay más complicidad quizás, o…**

Sí, nosotros en ese aspecto, no, no hemos perdido nunca nada

**Mmhum (afirmación)**

Son muchos años juntos ya y no, no hemos perdido nada. Y muchas veces a lo mejor empieza a hablar: ¿te acuerdas de jóvenes cuando esto y lo otro? No sé qué… me dice a lo mejor: “Ah! Igualito que ahora!” Bueno, y qué? Hay que ir adaptándose a las circunstancias.

**14:37 Claro.**

Pero ya está.

**Pero esa es una mentalidad muy buena.**

Sí, nosotros, en ese aspecto… a ver si de vieja eres capaz de mantener el ritmillo que tienes ahora… [Risas] que me vas a matar de viejo!! Y al final pues mira la pobre… está “fallando” ya

**14:54 Bueno… pero todo eso son cosas que se pueden…**

Sí, yo me lo tomo… ella se ríe y … con los amigos pues, pues nos reírnos y tal…

**15: 07 En realidad, la entrevista, básicamente, es esto…**

Entonces ella te ha contado más cosas entonces [Risas]

**No, porque ya hemos empezado a hablar de más cosas, pero vamos, lo que es la estructura de la entrevista, más o menos, era esa, pero luego, lo que sí, ehm, que hay… ay! Bueno, se me ha olvidado quizás, ay, un par de cosillas.**

Pero bueno, pregunta.

**15:27 Por ejemplo, hay muchas pacientes que dicen que la medicación le afecta mucho a nivel…**

Yo creo que sí…

**A nivel eh…**

…… y antidepresivos la libido es una de las cosas que te la apaga, por eso te decía yo que el otro día de lo del cese de las pastillas

**Claro.**

Pero claro, no me voy a poner a decirle, ella lo sabe, pero algunas veces lo hemos comentado y tal, digo, es que a lo mejor, entre todas las pastillas que te tomas y tal, resulta que tienes un cocktail ahí dentro que a lo mejor tiene poca libido, vale, pero es quela poquita que podrías tener después de una menopausia, pues qué quieres que te diga? Pues te la quita. Y los antidepresivos es una de las cosas que más te quita la libido y llevas años tomándotelos, digo porque después tú te pones a… te escitas y tal, y, y, y, y te excitas! O sea, no vas a lubricar igual que cuando tenías 20 años, pero bueno, se excita. Independientemente que ahora, a lo mejor con la con la…. Que tiene dentro, pero está bien, está bien. Que puedas usar más lubricante o menos, a lo mejor, depende del momento, a lo mejor dice: “Echa un poquillo y tal”, porque es normal, no lubrica igual que antes, pero si, uhm, estás tomando tantas pastillas que te cuartan todo el tema, pues dice uno, bueno, es que entre una cosa y otra… se junta todo.

**16:40 Claro.**

Pero bueno. Claro, ahora dile tú que se quite las pastillas.

**Bueno, también si le va viendo, si van yendo bien otras cosas alternativas y va viendo que ella se va encontrando mejor…**

Ya, ya, ya…

**Quizás, en algún momento… si ve que…**

Yo por eso… yo se lo dije el otro día, digo, es que tendría que ir, mira, decirle a Nicolás: “Mira Nicolás, me voy a quitar todas las pastillas, a ver cómo voy” Y conforme yo vaya viendo, si veo que esto me molesta mucho, pues me empiezo a tomas estas. Porque, a lo mejor, estas te las has quitado y sigues igual que ahora. Tú qué sabes?

**17:10 Es que esas ideas, por ejemplo, están muy bien, porque es una idea que cae, y cuando ella esté preparada, si está preparada y quiere, la coge…**

Ya, ya, esto ha sido una cosa que no la puedo obligar. Para que después diga: “Ay es que tengo dolores!” Pero es que lo dolores te siguen doliendo te tomes la ::: pina, te tomes una cosa, te tomes lo otro, sigues teniendo dolores! Te alivia de algo? La pastilla para dormir, se le olvido. Ha dormido? Sí! Entonces, no te hace falta! Otra cosa es que me digas: “es que me dieron las 4 de la mañana y estaba con los ojos como platos.

**17:39 Comiendo techo, claro.**

Vale. Es que no tomo ninguna pastilla. Paracetamol cuando me duele la cabeza.

**Claro.**

Pero yo de tratamiento crónico, es que no toma nada. Nada. Nada.

**Claro.**

Dejé la arcosia, que tengo un tobillo hecho polvo de una operación de hace muchos años, y me duele, me duele muchas veces. Empecé a tomar colágeno con magnesio y llevo, el arcosia llevo años sin tomarme una pastilla. El colágeno ahora, últimamente, también lo he dejado, porque llevaba mucho tiempo tomándolo y ya lo que hago es aguantarme.

**Claro.**

Porque sino ¿qué voy a estar tomando antiinflamatorios toda la vida? ¿Para que me haga polvo el cuerpo? Pues me aguanto.

**Es que eso a nivel hepático tampoco es bueno.**

Digo, mira, cuando hago un esfuerzo más de la cuenta pues me duele un poco el tobillo, al otro día me levanto nuevo. Pero esa tarde, en el momento en que me enfrío, empiezo a cojear, hasta que calienta el pie un poco, pero, el, ya está. ¿qué voy a hacer? ¿Tomarme la arcosia? No me tomo la arcosi, no me tomo nada.

**Es que estamos acostumbrados a que una pastillita nos cura, no sabemos…**

A mí me duele la cabeza y me tomo un paracetamol, porque sé que me va a más, y un paracatamol me lo corta, o un ibuprofeno, o una aspirina, me da igual, me lo corta ¿por qué voy a dejar que me duela la cabeza si hay una pastilla? Y además, no esoy todos los días tomándo pastillas.

**Claro.**

Ni la tensión, nada, lo tengo todo bien.

**¡Qué bien!**

Entonces, no tomo pastillas de ninguna clase.

**Y, por ejemplo, hay medicamentos que a las pacientes, yo creo que no es el caso, porque la veo muy bien, y muy, pero hay pacientes que se hinchan mucho, o medicación que las hincha mucho.**

Ella, es que últimamente le digo, porque yo le decía: “Lourdes, es que no bebes agua. Bebes muy poco” No. Es que no orinas, estás bebiendo más agua y no orinas, digo: a ver si tienes un poquillo de retención. Entonces vinimos el otro día, estamos hablando de la semana pasada, y Nicolás tocó y dice, tienes una poquilla de retención de líquidos. Entonces le mandó unas pastillas y se tiró toda la mañana orinando. Pero está bebiendo más agua que antes. Es que le digo tú es que, es que antes bebía poca agua, antes bebía poca agua pero es que ella podía ir al servicio por la mañana, a mediodía y a lo mejor por la tarde y después se acostaba y no iba al servicio. O sea, yotengo la costumbre de siempre antes de ir a dormir, orinar, porque me acuesto más tranquilo.

**Claro, no hay que levantarse por la noche…**

Así no me tengo que levantar y a ella le daba igual….

INTERRUPCIÓN DE LA LIMPIADORA….

YA SE ME HA IDO…

**Sí, de la retención de líquidos.**

Eso. Entonces le ha mandado la pastilla. Por la mñana se la toma y a medio día, tu estás largando ya. El anillo ya le va mejor y tal. Es que antes le venía… en juno, cuando se casó el major, “Ay qué gordita estás!” Estaba hinchada porque vino y le dijo: Nicolás, mandame algo que se casa el niño, algo que yo aguante los dolores.

**Es que esa es la medicación que se queja la gente, claro.**

Dice: algo que me aguante los dolores para aguanrtar el tirón de esa noche. Y le mandó un antiinflamatorio bastante fuerte y claro, parece ser que también la hinchó un poco.

**Mmm, tienen corticoides algunas…**

Y… Ay qué gordita estás!! Y ella no estaba gorda, estaba hinchada, estaba hinchada. Y después le bajó un poquito, pero le decía, estás bebiendo mucha agua y sigues sin orinar. Dice, ya, si eso es. Bebía más agua, por la tarde. Ahora me levanto y es: táreme un vaso de agua, y se lo llevo,. Y ya bueno, ahora si por las mañanas pues se lo está tomando. Tu ves, si nos vamos por ahí no se la toma.

**Claro.**

Si no no veas, vamos a estar cada dos por tres. Y el sábado igual. El sábado no se la tomó.

**Sí, pero aunque no sea todos los días… pero llevándolo controlado…**

No, no quiero que sea una cosa depor vida, cuando tu veas que ha aflojado, pues paras. Es una cosa que puedes tomarla cuando quieras.

**Claro. Eso sí está muy bien. Porque es importante. Es que, por ejemplo, hay muchas pacientes que dicen que al tomar la medicación, yo creo que es esa puntualmente, que se sienten muy hinchadas, y que claro, la autoestima también se veinfluenciada. Lo que pasa es que yo creo que si ella no toma esa medicación…**

A ella sí se le ha venido abajo. Y todo el mundo, cuando se arregla: “Ay! Qué guapa!” Además de verdad. Cuando la ves arreglada, pintada y tal.

**Es muy guapa, es guapísima.**

Hija, qué guapa estás, me están entrando unas ganas de… y dice: “No vayas a empezar ya!” Y… pero claro, ella lo sabe y todo el mundo se lo dice, es que ya quisieran muchas el tipo que tu tienes.

**Sí, es muy guapa.**

No sé si te lo ha dicho que tiene 53 años, el tipazo que tiene. “Lourdes, tu es que no te ves?” Ah! Pues no sé qué, pues no sé cuántos! “Qué te mires en el espejo, que nunca te has valorado!” ¿Tú te estás viendo el cuerpo que tienes cuando hay niñas que tienen 18 años con una celulitis en los muslos, y tal y los pechos caídos, ya quisieran muchas! Claro, a ella le encanta que se lo diga. Ella sí, como a todas las mujeres, es lógico.

**Claro, claro.**

A todas las mujeres que se lo digan. Pero la tiene muy apagada. Entonces hay que darle, darle, darle, entonces ella cuando se vea arreglada qué guapa. Claro, está guapísima. Pero te tienes que ver tú, te tienes que ver tú.

**Claro. Sí, pero eso, yo creo que es muy importante eso, la conexión, que tienen y la comprensión y la comunicación.**

Sino no llevaríamos 30 años juntos casados, más dos de novios, 32 años.

**Pero eso es super importante y no todo el mundo lo tiene porque en estudios anteriores la queja de muchas pacientes era esa, el, sí, mi principal apoyo es mi marido, pero hay veces que no me entiende, pero, por ejemplo, cuando se encuentra una pareja con buena comunicación, no, nosotros no tenemos secretos, es una maravilla, porque esto es lo que queremos ver, el qué funciona en estas parejas, el…**

Sí, pero yopienso que para que funcione tiene que haber confianza, si no hay confianza, si yo le estoy diciendo si yo te puedo, sí, sí, y luego te dice ella: ¡Que´va! Si este coge y se va todas las noches se vapor ahí y yo no sé ni a dónde se va.

**Claro.**

Entonces de qué te vale decir “yo me llevo bien con mi mujer, le digo, le digo, y luego llegan las 8 de la tarde y le digo: “me voy a dar una vuelta, que me “aflicho”.

Eso hacen muchos. Voy a dar una vuelta y se va de putas, se va de bares, se va de esto, se va de lo otro. Yo llego a mi casa, no es por ponerme bien, pero nunca he sido una persona de bares, como mucho, a lo mejor, entro en un bar a desayunar pòr las mañanas cuando estoy trabajando y salgo, pum, fuera, pero que como muchos. Sí, mi marido… si es que como yo le digo, yo tendría que haber sido malo, porque están las tías estas que están los tios borrachos estos jugando, “Ay! Mi Manolo! Y resulta que “El Manolo” es un “putañero”, se lo gasta todo en el juego, en vivio, en el bar y digo, lo quieren con locura. Y uno, que está metido en la casa y no hay más que p`roblemas.

**Qué va, hombre. Qué va. [Risas] Pues yo creo que está muy bien la comunicación y la comprensión…**

Sí, yo creo que es lo principal. Si hay confianza. Yo paso. La semana pasada, se encontró donde vivíamos antes, en La paz, a un amigo nuestro de la pandilla, y le dice: “oye, tu te casate con Emilio, ¿no?” Sí, claro. Y sigo casada. ¿Y sigues casada todavía, porque es que de la pandilla…

**No queda nadie, claro.**

Mis compadres y nosotros, somos los que todavía estamos casados. Los demás se han separado TODOS, todos, todos. Pero estás todavía,pues no veas, llevais un puñado de años, y dices, sí, y espero que queden más, porque es que dices, es que yate extraña porque tú le dices a un chaval hoy que llevas 30 años, y dice, Uy! Tú estás loco.

**Bueno, en mi generación se están separando con un año. Muchos amigos que tengo…**

O vienen ya separados del viaje de bodas [Risas]

**Digo: Yo a esa boda no voy que eso no va a salir rentable. Yo, sí se quieren de verdad sí voy, sino no.**

O no se toleran, o no aguantan. Por eso te digo es que no aguantan o no se han conocido lo bien que se tenían que conocer, mo lo entiendo.

**Puede ser. Tantas cosas.**

Pero es que no lo entiendo si habiendo confianza. Ahora, tu sabes lo que pasa? Yo pienso que también porque, a lo mejor antes la mujer tarabajaba menos, se adaptaba más al matrimonio, a la pareja, y tal, pero ahora con la cosa de que cada uno tiene su independencia y lo veo normal. Y bueno, yo porque tengo que estar aguantando a este o a esta si yo puedo estar por mi cuenta? Es que claro, a la mínima, salen pitando uno y otros.

**Sí pero yo creo que tiene que ver con lo que ha dicho antes de conocerse bien de verdad, estamos acostumbrados a conocer un poquito, nos gusta y ya y lo damos todo, no hay conexión de verdad.**

Cuando convives, es cuando tú te das cuenta de que, de si funciona o no funciona.

**Claro, y esa conexión, muchas veces a la p`rimera de cambio, no dejas la, estamaos acostumbrados a usar y tirar. En cambio, si esa conexión está ahí, y eso es fuerte de verdad, auqnue haya problemas, se puede ir quitando la porquería y decir: esto es lo importante, venga, seguimos.**

Sí nos ha pasado, hemos tenido problemas como todas las parejas, más achuchados económicamente, mejoría económicamente, achhucahmiento, crisis, de todo, hemos pasdo por todas las etapas, pero ahí va, va y va. Vas toreando, vas toreando, vas toreando y la vas llevando.

**Claro.**

Y lo vas llevando porque estamos los dos a una.

**Claro! Ahí va! Es la misma sintonía.**

Claro, y si tu empiezas a tirar de un lado, el otro tira del otro, y pues mira, vete con tu madre que ya mañana nos veremos. O vete… y al final no se aguanta. Pero si vas todo a una, esto es una empresa.

**Un equipo, claro.**

Y tenemos que estar los dos sociosen una, sino no funciona.

**Todo para una.**

Entonces claro, que pasa? Ella siempre ha sido muy negativa, yo he sido siempre muy positivista y yo creo que eso es lo que ha sido que nos ha complementado.

**El equilibrio.**

Nacho, cuando era pequeñillo, que nos dio muchos problemillas también de chico, tenía un :…………..: roto, tenía esto tenía tal, y ella estaba hundida, y digo, digo Lourdes, que hay que tirar palante! Me llegó a decir un día que si yo no quería al niño. Claro que lo quiero, igual que tú. Digo, pero si yo me hundo igual que tú, ¿quién tira del carro palante?

**Claro.**

Y ya está. No hubo más conversaciones del tema. Digo, sí yo me hundo igual que tú, ¿quién tira, quién tira del carro?

**Sí, pero ese equilibrio es muy importante en la pareja.**

Hombre, si somos los dos positivistas no hay problema.

**Mmmm, a veces si, eh?**

Sí, pero si los dos son negativos

**Sí, siempre tiene que haber un equilibrio, porque silos dos son muy positivos, entonces no se hace nada porque no hay ningún problema.**

Ya, ya.

**Y hay veces que los problemas así, es cuando se hace una bola grande.**

::::::::::.. Pero yo veo las cosas más de esto, me tomo las cosas de otra manera, y verás tú, a lo mejor me puede afectar, cuando estoy muy nervioso, empiezo, pin-pon, pin-pon, pin-pon, pero, a lo mejor exteriormente, no lo… no lo aparento. Un par de veces me ha dado unas taquicardias y tal, esto lo mismo es del estrés, me han hecho un electro y tal, pero bueno, el estrés, sí, pero es que tampoco sabe uno si es del estrés o ha sido una cosa puntual o dices, si tienes mucho estrés al final te va a salir por algún lado,

**Por algún lado, claro.**

Entonces pues… así lo vamos llevando y ya está.

**Pero muy bien.**

Sí, dentro de lo que cabe lo intentamos llevar lo mejor posible.

**Pues, si quiere, lo podemos dejar aquí ya, yo creo que está todo.**

**¿CUANTO HACE QUE LE DIAGNOSTICARON FM A SU PAREJA?**

No me acuerdo, hace ya tiempo, pero no me acuerdo ya. No te puedo contestar por que no me acuerdo, hija.

**SE ACUERDA CUANDO FUE LA EPOCA DEL CAMBIO EN CASA, DE DECIR TIENES FM**

Cuando ella empezó con los cambios fue cuando a ella empezó a retirársele la regla y eso pero vamos que yo he ido siempre con ella al médico allí en Sevilla, de lo de la FM no te puedo dar fecha, no te la puedo dar, el darte una fecha es engañarte, es que no me acuerdo, si me acordara… El decirte por ejemplo hace 5 o 6 años, o 7, es engañarte. No sé si hace más o menos…

**SI NO TIENE QUE SER PRECISO, ES SIMPLEMENTE PARA PONER EN CONTEXTO**

Si, pero no me acuerdo, si me acordara… Es que ella ha estado liada con el esófago en el Macarena, allí en Sevilla…es que no me acuerdo, cuando le dijeron lo de la FM es que no me acuerdo, vaya que haga 5 o 6 años, o 7 más o menos…

**NO ES UNA PREGUNTA MUY IMPORTANTE TAMPOCO**

Sí, ella si lo sabe porque lo tiene ella, pero yo no te lo puedo decir fijo, no te lo puedo decir.

**POR EJEMPLO: ¿CÓMO CAMBIÓ LA CALIDAD DE VIDA, POR EJEMPLO?**

La calidad de vida para ella ha cambiado bastante, jejeje, desde que yo me prejubilé y nos vinimos aquí a Málaga, la calidad de vida ha cambiado bastante, un 100%, hombre por Dios, hombre por Dios…

Ella es más nerviosa que un rabo de chivo y estando ella no más aguantando para arriba y para abajo… porque ya hace 3 años que hemos venido.

Pues le ha cambiado la vida, le ha cambiado la vida mucho…

**¿USTED COMPRENDE LO QUE ES LA FM?**

Hombre, yo si la comprendo a ella porque hace 3 o 4 días ha estado con la lengua blanca. Porque ha estado en el dentista y le ha dicho que usted sabe que la FM se le sale a la lengua y… no lo sabía. Hace cuestión de una semana que le dijeron. Al lado d la lengua se le puso blanco… ella como come fruta… digo… vamos a dejar la piña… que ya para que obre mejor y eso…

Y digo, vamos a dejar la piña, vamos a descartar la piña y ahora vemos con otra.

Ahora dice: será de los caramelos que yo me tomo por la tarde, que sabe, lleva comiendo caramelos 6 o 7 años y ahora se pone y le va a echar la culpa a los caramelos, pues yo creo que no, entiendes lo que

**CLARO, HAY QUE BUSCAR LO QUE PUEDE SER…**

Lo que puede ser es que a ella le han dado unos brotes de FM y le ha dado fuerte, le ha dado fuerte y yo poniéndole a lo mejor los pañitos, las mantitas, esas eléctricas y eso, de calor, dándole calor aquí y sobrellevarla, que le vamos a hacer.

Si el médico a nosotros nos dijeron allí en Sevilla, nos dijeron, mira, la FM es: el día que estés buena aprovéchalo y el día que tengas FM tendrás que aguantarte.

**CLARO, ES DIFÍCIL DE ASUMIR**

Entonces, eso dice que se lo dijeron, el médico, eso es…

**CLARO, ESO ES…**

También ella tuvo psoriasis, no se llama psoriasis? Nos mandaron a que se fuera mucho a la playa, que pisara las algas, porque le salió aquí, en la `plante de los pies y se le quitó, se le quitó…

**SI, EL YODO QUIZÁS**

Sí, sí, el yodo de Chipiona, dicen que era muy bueno, que pisara las algas todo lo que pudiera y ahora parece que le ha salido no sé dónde una mijilla, pero nada, es muy poco, muy poco, gracias a Dios es muy poco.

**PERO VA MEJORANDO**

Sí, si le ha dado un cambio en la vida que no veas…

**Y POR EJEMPLO LAS TAREAS DEL HOGAR?**

Las tareas del hogar la hago yo. ¿SI?

Hombre, a ver si me comprendes, las hace ella, pero todo lo que pueda ayudar, ayudo.

**QUÉ BIEN**

Planchar, no plancho porque no sé planchar y la cama, dice, Manolo, vente p acá que vamos a hacer la cama entre los dos. Le friego, pues todo lo que le pueda quitar de golpe, se lo quito.

**PUES ENTONCES COMPRENDE MUY BIEN…**

Pues claro, desde los 14 años juntos, tu fíjate, imagínate. Le digo tu que pasa, que no te echas cuenta de lo que yo hago? Y dice – Bastante tiempo lo he hecho yo, ahora lo echas tú.

Claro, nos compenetramos muy bien, muy bien.

**¿Y CÓMO HA INFLUIDO EL DIAGNÓSTICO DE FM EN LAS RELACIONES DE PAREJA?**

En las relaciones de pareja han sido bien.

Lo único que pasa es que a ella, al retirarse la regla ya está, según me dice a mi ella, yo no entiendo de eso, dice que está muy seca por dentro y le da miedo y dice que le da miedo, le da miedo porque dice que le duele mucho y hace bastante tiempo, no bastante tiempo, no, tampoco mucho tiempo, pero es que le da miedo… y yo qué hago…

**¿HA PENSADO EN UNA ALTERNATIVA, MASAJE U OTRAS COSAS QUE SE PUEDAN HACER PARA TENER INTIMIDAD?**

Nosotros cuando nos acostamos, nos acostamos a dormir. Nosotros nos acostamos muy tarde, desgraciadamente nos acostamos muy tarde, bueno, afortunadamente nos acostamos muy tarde con la televisión y nos levantamos muy tarde y nos acostamos muy tarde y cuando nos damos cuenta decimos ¿Y ahora qué pasa?

Con el frío que hace cualquiera ahora se destapa, jajaja y con el frio que hace cualquiera se destapa… y para poner el culo afuera ahora, jajaja

Y no se puede poner el calefactor porque la luz está como está…le digo mira, a joderse

**HASTA MAYO NADA**

Hasta que no llegue el veranito que se pueda uno destapar, cualquiera… no saca uno el culo

**Y LUEGO CON 40 GRADOS..**

Con el calor se va uno a la piscinita o se pega uno un duchazo, un duchazo mientras termina o no termina

Cuando vaya a terminar ya viene el calor pero antes no. Pero ahora, ahora para sacar el culo tienes que tener cojones, jajaja y con todo los edredones y todo lo alto cualquiera…jajaja

Te voy a decir otra cosa, que cuando se lía uno le entra calor, claro.

**ESO SI ES VERDAD**

Claro… pero a ella le da mucho miedo, ahora mismo lo del sexo le da mucho miedo, bastante miedo y yo como la respeto digo ahora que vamos a hacer…

Desde los 14 años, como le han dicho si es que, ostras si es que estás desde los 14 años, me lo tienes exprimido, es que estoy desde los 14 años con ella eh. Yo tengo ahora 64, fíjate. Y ella cumplió ayer 57.

**PERO ESTÁ BIEN LA RELACIÓN, LA COMPRENSIÓN…**

Si, la verdad sea dicha, en todos lados, bueno, en todos lados. Hasta mi hijo me dice… me dice… yo quisiera llevarme con mi mujer la mitad que lo que mi padre con mi madre y ya ves que afortunadamente se lleva muy bien con su mujer.

**QUE SUERTE**

Si, si en serio. Nos tienen mucha envidia la gente. A mí me da igual. Qué envidia, qué envidia. Hay que ver tu Manolo cómo le tiene. Pero esto es lo que hay. Es lo que ha tocado, pues es lo que ha tocado.

**SE LO MERECEN LOS DOS**

Tosa la vida trabajando más que un mulo. Pues ahora me ha tocado vivir la vida, por lo menos un poquito.

**¿Y DESDE QUE ESTÁN AQUÍ ESTAN MEJOR, NO?**

De maravilla, de maravilla, bueno, es más, mira…

Yo llevo 3 años viviendo aquí, 3 años aquí, pero nosotros tenemos el apartamento desde hace ya 12 años, y ella, cuando se estresaba cualquier cosilla, digo yo shhh pa allá

Nosotros veníamos aquí todos los fines de semana. Digo, tú te quedas aquí, que tu hijo está aquí también. Yo me voy para allá, trabajo y en el fin de semana me vengo para acá. Yo me la dejo aquí muchas veces.

**Y ESO LE SENTAB BIEN**

Hombre, fíjate, ya ves, levantarse e irse a la playa, a quién no le viene bien eso? Jejeje

**QUÉ BIEN, A VER SI SE ME OLVIDA ALGO MÁS, AY SÍ, POR EJEMPLO, A UNA PAREJA QUE A SU MUJER LE HAN DICHO QUE TIENE FM.**

Yo, que lo sobrellevara como ella lo sobrelleva, otra cosa qué le voy a decir?

**¿QUÉ CONSEJO LE PODRÍA DAR, DE HOMBRE A HOMBRE? ¿QUÉ LE DIRÍA?**

Hombre, que la mimara un poquito, que la mimara un poquito. Que tuviese más cuidado con ella. Quitarle todos los golpes que se pueda. ¿Sabes lo que te quiero decir, no? Y mimarla, que le vamos a hacer. Si, es la que tenemos.

Mientras sea FM, que todos los males fueran esos.

**PUES MUY BUEN CONSEJO, LO DE MIMARLA, QUERERLA, COMPRENDERLA MUCHO.**

Yo hay veces que digo, hombre, no pasa nada, cuando pasen 2 horas se te quita, tomate una pastilla y tal. Yo lo digo por callarla, y te digo una cosa, si quieres que se te quite eso, te tomas dos whiskys, te tomas 2 whiskys y se te quita todo.

**EL SENTIDO DEL HUMOR ES MUY IMPORTANTE**

Hombre por Dios, hombre por Dios. El sentido del humor es lo mejor. Eso es lo mejor. Ella te ha dicho que se está tomando, como, no se lo que le han dado…

Te he dicho que una vecina mía, que es ATS, y el otro día estuvo comentando, y le dijo la vecina, tómate esto y le ha dado resultado.

Pero quiere decírselo a Nicolás, a ver si le dice que si eso es, como se dice que es, no me acuerdo la palabra… no vaya a ser que sean corticoides y quiere abusar de ellos, sabes lo que te quiero decir…

**SI, PORQUE ELLA ME HA DICHO QUE SOLO TOMA ANTIINFLAMATORIOS, HAY MEDICACIONES QUE SIENTAN REGULAR**

Por eso le tiene que preguntar a Nicolás cuando eso…

**HAY MEDICACIONES DE FM QUE A VECES SIENTAN…**

No, no, no

A veces tiene brotes de FM. Afortunadamente ya te digo. Afortunadamente tiene poquito. Si estuviese la criatura todo el día en el suelo de… o en la cama, de dolores y eso… pues mira, habría que tomar otras medidas y eso… pero…

A lo mejor le dan 3 días y no fuerte-fuerte. A veces le ha dado algo fuerte, pero al otro día…. A ver hombre, claro. Lo tiene controlado, gracias a Dios, gracias a Dios. Si.

**PUES, EN REALIDAD HEMOS TOCADO TODOS LOS PUNTOS IMPORTANTES, LA COMPRENSION, LA COMPRENDE PERFECTAMENTE.**

Ya puede entrar ella?

Oiga señora, venga usted para acá, venga usted para acá señora. Dice la muchacha que si yo te comprendo a ti, hija…

**Hace cuánto tiempo más o menos le diagnosticaron la enfermedad a su pareja?**

Pues yo creo que hará un año y medio o un año y ocho meses o así, creo, ¿Eh?

**Y desde entonces, ¿ha sido un cambio muy radical la, por ejemplo, la vida en pareja? ¿La convivencia? ¿La calidad de vida?**

Ha cambiado un poquito, un poco, no mucho tampoco, no sé crea, pero ha cambiado algo.

**Sí. Y a la hora de, por ejemplo, reparto de tareas o en el hogar y eso…**

Coopero muy poco. Ahora coopero algo más pero siempre he cooperado muy poco. Le he echado todas las tareas a ella prácticamente. Como ella las ha hecho y los hombres muchas veces pues…lo dejamos, lo dejamos y ella, aunque parezca que no es una mujer dura, pero yo sé que muchas veces no puede con las tareas…

**Claro.**

Y le cuesta trabajo, pero ella lo hace.

**Claro. Y , ¿comprende usted lo que es la FM? ¿Comprende…?**

Creo que sí, creo que sí. He estado leyendo cobre ello, he escuchado programas de radio muy interesantes, cuando han tratado el día de la FM pues he estado muy atento, he buscado en internet, comprendo perfectamente.

**Y ¿la entiende cuando un día no puede?**

Claro que la entiendo. Claro. Que me duele, pues claro, pues claro, no te va a doler?. Pues si te duele. Y no lo disimules que yo sé que te está doliendo ahora. Es así, y ella sí tiene dolores, cosas que se calla, claro, cosa que se calla, pero que yo lo noto pues en la manera de dormir, ronca más, se mueve, mueve la pierna.::::::::::::::::::::

**El descanso no tiene tanta calidad, no?**

El descanso no tiene mucha calidad. No, (chchan) no, exactamente. Es una de las cosas que yo :::: me ha llamado más la atención. Ella antes se levantaba, pues no con más ánimo pero con más viveza, un, con una viveza con otro, con tono de cara, con una sonrisa, y ahora pues… y disimula algunas cosillas, es de dolor. Eso sí me doy cuenta. Es que es obvio vaya…

**Si hay días y días, ¿no? Hay días que se nota mucho…**

También, sí, exactamente. Hay días y días, exactamente.

**Y a nivel de, por ejemplo, un día especial, como calidad de vida, en plan de un día que nos vamos a hacer algo más especial, una excursión o un viaje, ¿cómo influye eso?**

Ella pues, pues ella lo agradece muchísimo. Pues vamos a poner del 1 al 10, pues ella digamos que cambia en un, en un 8, y yo pues también estoy más feliz, en un 4, que tampoco yo soy muy positivo muchas veces, pero yo lo noto. Y es bastante, me hace bastante feliz que ella esté bien también.

**Y, por ejemplo, a nivel de la relación de pareja, ¿cómo ha cambiado desde la FM?**

¿La relación de pareja sexualmente?

**En todos los niveles.**

Ha cambiado algo. Pero siempre hemos tenido más o menos una misma línea, una misma línea. Que la línea está un poco más descendente, no sé ha notado porque ha sido el pico muy poco acusado, digamos. Algo sí ha cambiado, sí.

**Y a nivel, por ejemplo, de relaciones íntimas, ¿ha cambiado?**

Bastante. Bastante, sí. Vaya, no sé si ella te lo habrá explicado pero tampoco yo he sido muy activo, activo sexualmente, ni ella tampoco, por lo menos con ella. Y ha cambiado algo. Ha cambiado algo.

**Y han intentado buscar alguna alternativa?**

Compensarlo con alguna otra cosilla, pues sí, sí, ella es muy feliz cuando yo la abrazo, o vemos una película juntos. Ella lo agradece muchísimo, incluso estando con mi sobrina, hablando con la pequeña, como le decimos, y hablando de sus cosas, de sus tonterías y que haya, con un poco más de comunicación que le exprese, ella, pues digamos, lo agradece muchísimo. Y me gusta pues seguir, tener feeling con ella, porque sé que así se siente bien. No lo hago tampoco muy asiduamente pero cuando lo hago, se nota. Estamos los dos mejor.

**Y a nivel de, por ejemplo, de, por ejemplo, de las personas que tienen FM agradecen mucho masajes y eso.**

Exactamente, ella de siempre ha agradecido mucho los masajes. Yo soy una persona que me llamo antimasaje, no me gusta que me dan masajes.

No me gustan, se me pone la piel de escarpia, o noto una tensión en la piel que no es muy agradable. A mí no me gusta que me toquen. Es más, me gusta que me hagan, a lo mejor, un masaje pero fuerte, que me, los músculos, que me estiren. Compañeros, no estoy en un gimnasio pero compañeros con los que corremos o hacemos, “espérate Miguel, te voy a dar un tirón en la pierna porque he visto que…” “venga, venga que…” Eso lo agradezco más que un leve masaje en la espalda, algo más rudo. Sin embargo, ella no. A ella le gusta más bien sentir la piel, la ternura, la caricia, y somos distintos. Como la noche al día en ese aspecto.

**Y es difícil, por ejemplo, conectar de esa manera a nivel de pareja, o…**

No, no, de momento no. Todavía se puede cvonectar bien en ese sentido, sí. Cuando ella me pide un masaje, yo le doy un masaje. Pero ella sabe que yo masajes los mínimos, [Risas]

También le pido, de vez en cuando, un masaje rudoy fuerte, porque me duele mucho pues la pierna o la espalda, y: “no, tú pega aquí, tú sigue haciéndolo fuerte pòrque no me vas a hacer daño”

**Claro, cada uno a su estilo, claro.**

**[Risas]**Exactamente.

**Y, por ejemplo, ha, otras alternativas que hayan encontrado como para seguir teniendo…**

Más, más sentido a la vida? Cómo darle más sentido o más…

**Más conexión en la pareja, tener más, no sé, como momentos para, de intimidad que no tienen que ser solamente sexual, sino intimidad en el sentido de decir, pues vamos a preparar esto para nosotros, como hacer un poco sagrada la relación.**

Em, bueno, por parte de ella sí, bastante. Una cena especial, un algo, un algo de decoración en la casa, un regalo que me hace o una sorpresa, también me da sorpresas, sí, se lo agradezco mucho eso, en ese aspecto muchas veces me sorprende con esas cosas a mí: “toma, que sé que te gusta, he hecho esto”. Y luego he hecho una tarta, he hecho algo especial para ti. Sí, ella lo disfruta, mucho más que yo

**Por hacerlo, no?**

**[Risas]**

Porque le gusta tanto la cocina.

**Claro.**

Lo que menos le gusta es después claro, los cacharros que se quedan por medio y demás, tener que limpiar y…

**Claro [Risas] los buenos chefs son así, no necesitan a alguien que lave.**

Exacto.

**Y, por ejemplo, ehm, si a una persona le acaban de diagnosticar FM, ¿Qué consejo de daría usted a su pareja? Como consejo.**

Como consejo que, que, que escuche la voz del especialista, que no se guíe por las tontería de que escucha en, en ciertas reuniones, personas que no son indicadas, que se, que tenga relación también con personas que tengan ese problema, para conectar, para intercambiar opiniones y que no lo deje y que si necesita ayuda por mi parte más, que me lo pida que estoy ahí también.

**Mmmm. Y a nivel de consejos de cómo llevar la pareja? ¿Cómo, qué le diría en plan…?**

En que plan?

**Por ejemplo, cosas que han servido para que su pareja siga unida que pueden servir para que esa pareja siga unida también, el decir, la FM llega a una casa y es como eso, como un tsunami, ¿no? ¿Y qué hago yo para poder comprenderla? O qué hago yo para no sé, para cuidar de mi de mi pareja, ¿qué consejo?**

¿Qué consejo…?

**Le daría usted.**

¿Qué consejo para cuidar, no?

**Sí, de decir, pues a mí me ha ido muy bien, mmm, no sé, comprendiendo muy bien a mi pareja, observándola o casas, sí.**

Sí, eso sería el primer punto, creo. Ese sería el primer punto. Observándola y escuchándola y estar en la piel, en la piel de mi pareja, eso sería fantástico.

**Empatizar, no?**

Empatizar! Efectivamente, exactamente, sí. Creo que sería la parte fundamental. Yo creo que en todas las enfermedades.

**Y algunas cosillas más así, algún truquillo, “cuando tiene crisis” [Risas] no sé, algún truquillo así, ¿Qué une a la pareja? Qué…**

Cuando tiene, pues no lo pienses, si puedes hazlo y sino, no lo hagas, hazlo mañana. Ya está, no te preocupes, no te comas tanto el coco porque posiblemente mañana estés mejor. Esto será una crisis. Ya está. Cosas así.

**Y qué consejos para alegrar a la pareja? ¿Qué?...**

¿Para alegrar?

**Para esos días que uno dice que, es que claro, es un dolor constante, 24 horas al día. ¿Qué, qué haría, qué consejo le daría? Pues a mí eso me ha funcionado estupendamente. Para alegrar el estado de, físico y mental de…**

Pues la verdad es que consejos doy muy pocos pero alguna vez que otra, pues no sé, si decir, si mañana es otro día, si hace un buen día, podemos andar los dos juntos.

**Mhum..**

Pasear en el coche, ir a los sitios que hemos ido anteriormente cuando éramos jóvenes.

**Claro.**

Cosas así, de ponerle un poco de salsa a la vida y olvidar la, los días malos. **Mhum, pues eso está muy bien. Muy buenos consejos.**

Sí, yo creo que sí, vaya. Ir al cine también. No, no no vamos mucho al cine, pero disfrutamos, disfrutamos cuando vamos al cine, las pocas veces que vamos pero lo disfrutamos. Y parece que nos olvidamos de muchos temillas, de muchos temillas, de la FM, parece que estemos como, que estás viviendo nuestra, nuestro reinicio de, de pareja.

**Ah! Qué bien!**

Cosas así, sí. Pasear, incluso, por ese pareo marítimo, que lo hemos hecho muchas veces. Ella es de Alharurín, yo soy de Málaga pero nos conocimos por aquí y es suficiente andar por ese paseo marítimo. Parece que hay una, algo, unas sensaciones especiales, no? Cuando andas por aquí.

**Una conexión de nuevo con…de cuando se enamoraron.**

Sí, una conexión, sí. Claro, claro ¿te acuerdas de esto? ¿Y te acuerdas? Y esto y lo otro?

**Y se evoca ese tiempo, no?**

Se evoca, sí, bueno, tiempos pasados. [Risas]

**Qué bien. Qué bonito.**

Eso sí.

**Pues son muy buenos consejos, son…**

Bueno, tampoco lo llevo mucho a la práctica pero de vez en cuando si, lo hago también.

**Y qué hace que no pueda llevarlo a la práctica?**

Porque soy una persona de unas ideas, no sé, no caigo en ese momento, no soy muy detallista, para cuando me acuerdo, pues sí, me gusta expresarlo también y si incluso ella quiere hablar de ese tema yo también lo hablo, no me opongo y no me niego y no, no, no, no lo hago porque no te lo mereces. No lo hago porque no me acuerdo y no soy una persona detallista. Por eso, pero cuando me lo recuerdan o estás en el momento de, de practicarlo, pues sí, sí y no me molesta, vaya.

**Claro, pues está muy bien. Pues quizás esa es la idea, que uno no cae porque está muy ocupado, estamos con una rutina…**

Pero ella me comprende, llevamos ya mucho tiempo, yo creo que ¿te acuerdas cuando esto? Y yo le digo, pues no me acuerdo, pues sí, pues sí es verdad! Y la pongo a prueba. No, pues fue así, la engaño un poco, no no así no fue, [Risas] claro…

**Pinchando un poquillo…**

Ya nos conocemos un poco, no?

**Claro, está muy bien.**

Bueno, se hace lo que se puede.

**Eso es muy importante, sobre todo eso, el…**

La unión, no?

**La unión y el que si caemos decirlo, o hacerlo o el cultivar el seguir cuidando la relación.**

Claro, claro.

**Porque para las personas que tienen FM sobre todos es que salía increíble el marido el , la pareja, es el apoyo más importante.**

El punto de apoyo, es un punto de apoyo muy…

**Entonces que la pareja sepa lo importante que es, es muy importante.**

Claro.

**Además, las herramientas que la pareja tiene, si las usa, son una maravilla, es que se, todo se pues de hasta mejorar, porque son días que dices: Ay! Estoy loca porque vamos a ir al cine mañana o vamos a ir a un Spá, o me va a dar un masaje o me, y esas cosas ilusionan mucho y hacen que la, que se mejore también.**

En ese punto, como no soy una persona tan mágica y no estoy siempre dispuesto, ay! Que esto qué maravilla, casi, a lo mejor, cuando, cuando lo hago…

**Es mucho más…**

Ella es, es más reactivo, es más… (Ruido) Interrupción y fin.

Cierran el centro, lo tenemos que dejar aquí.

**P: ¿Hace cuánto tiempo le diagnosticaron de fibromialgia a su mujer?**

E: Yo pienso que hace ya más de 10 años.

**P: Más de 10 años ¿y cómo fue la noticia? ¿Cómo llegó la noticia a casa?**

E: No ella se fue a una asociación de aridio porque ella se notaba que le pasaba algo pero no sabía lo que era. Entonces mi mujer es la típica persona que le da a las 3-4 de la mañana y ha estado pues limpiando la casa y haciendo esto y haciendo lo otro y preparando lo que hay. De momento está totalmente agotada. Y eso es algo chocante. Y ya cuando supo que pudo ser esto, pues fue un alivio para ella por lo menos saber lo que era.

**P: El ponerle un nombre muchas veces ayuda mucho.**

E: el ponerle un nombre sí.

**P: Y desde entonces como afecta por ejemplo a una vida diaria en casa?**

E: Pues es difícil, es muy difícil. La enfermedad esta tiene brotes, y son brotes muy muy muy fuertes. Por lo menos, hablo de mi mujer de lo que conozco.

**P: Sí**

E: Y ella tiene un trabajo físico. Entonces, el trabajo físico son 8h de trabajo físico. Por lo que yo he leído y he indagado la fibromialgia en comparación es como si te cargas una mochila de 50kg y vas a 50º por el desierto. Que es muy muy pesada de llevar, puedes hacer las cosas pero tienes una fatiga muy muy grande. Si a la fibromialgia se junta con el tema de la fatiga crónica entonces una persona hiperactiva como puede ser mi mujer, pasa a ser una persona pasiva totalmente. Entonces es difícil, muy difícil.

**(2:14)P: Un cambio muy radical**

E: Y luego la fibromialgia, el otro hándicap que tiene son las relaciones sociales.

**P: Claro**

E: En las relaciones sociales, tú no puedes hacer ningún plan. Que decimos a quedar dentro de dos semanas que nos vamos para tal sitio, porque dentro de dos semanas puede que tenga un brote y no pueda ir.

**P: Claro**

E: Y pues…

**P: Claro. Esa era la siguiente pregunta. ¿Cómo se vive…**

E: Ah bueno.

**P: No no estupendo, porque los dos me están respondiendo a preguntas sin hacerlas. Entonces está genial ¿Cómo se vive un día extraordinario, un día que se haga un plan como una boda, una cosa así pero si es lo que ha dicho es eso que…?**

E: Pues mira te voy a decir por ejemplo, un plan extraordinario. Por ejemplo cuando mi hija hizo la primera comunión, que fue en el 2009. En el 2009, una de las cosas que teníamos planificado para que ella tuviera toda su vida un recuerdo de ese día, fue que nos fuimos a Eurodisney 5 días. Cuando fuimos a Eurodisney le dio un brote de estos. Pues entonces tú imagÍnate el plan de que estás a no se 2000km de tu casa en un parque temático, con tu hija totalmente ilusionada y tú tienes que estar en el hotel porque no puedes con tu cuerpo.

**P: Pobrecilla**

E: entonces pues para nosotros fue duro, para mi niña y para mí, pero supongo que para ella mucho más

**P: Mucho más claro. Y eso por ejemplo, ¿Cómo es la comunicación con la familia? Cuando surge una crisis, ¿cómo se gestiona a nivel de pareja o a nivel familiar?**

E: Pues mira, nosotros tenemos una hija, entonces mi hija me mira a mí. Mira mi comportamiento.

**P: Claro**

E: Ummh… Cuando le da la crisis fuerte, no no es dueña de su cuerpo. Entonces o se mete en el sofá o se mete lo que es la cama y no hay?¿¿?. Y ella aunque le decimos que no pasa nada y que comprendemos lo que es, ella se da mucha coartada a la cabeza y que “si soy una inútil, que tal, que pa’arriba que p’abajo. Entonces nosotros intentamos de cuidar. No sé es duro. Tanto por parte de mi hija como por parte mía.

**P: Sobre todo verla sufrir y ver que…**

E: Sí

(4:43)**P: Y a nivel por ejemplo de pareja ¿cómo influye en UN día a día?**

E: Pues te cuento P. A nivel de pareja hay q tener muchas muchas luces. Yo te comento, yo por ejemplo estoy en la década de los 40. Tú todavía no has llegado aún a la década de los 40. Cuando llegues a la década de los 40, te darás cuenta de que es la mejor década con diferencia. Porque físicamente estás bien, económicamente lo llevas “medio bien”, porque los hijos te dejan un poquillo de tiempo libre, que ya están crecidos y entonces puede ser un buen momento de por ejemplo de disfrutar. Entonces es chocante. Porque yo por ejemplo hay veces que pues viajes y tal…

A nivel sexual por ejemplo, comentando los hombres somos hipersexuales, somos como los gallos y no me acuerdo de la última vez que tuve relaciones con mi mujer.

**P: Claro**

E: Entonces pues bueno, pues lo llevamos.

**(5:57) P: Claro y por ejemplo alternativas a una relación sexual normal, por ejemplo masajes, o incluso ir a un spa o algo así que sea un poco más como contacto pero quizás no tan sexual, ¿han intentado por ejemplo alguna alternativa?**

E: Lo he hecho de mucho de menos P. Yo se lo comento a mi mujer. La relación sexual no es solamente el coito.

**P: Claro**. **Es eso**

E: No es el coito. Pero también tienes que comprender P de que si yo lo comprendo a ver si me entiendes. Si yo tengo un dolor de muelas grandísimo, por muy bien que esté mi pareja, a mí lo último que se me pasa por la cabeza es…

**P: Claro. Total**

E: Y eso es así. la pareja los dos sufrimos. La pareja lo sufrimos y mucho. Muchas veces si no es. Yo a mi mujer le digo, que un día que estés bien. Acuérdate que tienes mareos.

**P: Claro hay que aprovechar jajaj**

E: No es cierto. Y a nivel confidencial te digo. Muchas veces si me ha pasado por la cabeza la separación o alguna cosa. Por la misma razón que te digo, es decir, tengo 40años estoy bien, y la vida pasa, y que pasa y ¿no voy a disfrutar nada? Le digo a mi mujer que llegará un momento que con la pastilla azul ¿¿?¿?¿ Es que no comprendes como estoy yo que tal y es verdad, que no puedo hacer nada. Y ese es el tema.

Pero bueno afortunadamente el amor puede más que todas las cosas. Que no deja de ser que me pase por la cabeza en algún momento de decir, más que nada cuando veo a amigos y tal, y ¿yo qué? yo también quiero.

**P: ¿Y esto por qué y para mí no? Exactamente lo que dices, que es justo una edad que ya está como todo en su sitio y se podría…**

E: No ya lo veras, ya lo veras P. Cuando llegues a la década de los 40, es una edad ¿¿?¿ a ver si me entiendes, y mi mujer me dice, “ y a tí ¿no te duele nada nada nada?” No se lo cree y dice, “pero bueno ¿nada nada nada?” Pues no, claro es que a ella le duele desde la uña del pie hasta el dedo... “No, no me duele nada”. Qué pasa que estás en una década que todavía estás bien de salud, que digamos estás posicionado laboral como económicamente, entonces pues voy a disfrutar. Quizás cuando me dé un achaque que tenga lo que esto y tal, pues entonces ya no podré hacer cosas, pero ahora sí puedo. Pero qué pasa con nosotros que ahora puedo, pero no puedo.

**(9:06)P: Claro, es como podría pero no se puede llevar a cabo. Y por ejemplo, a nivel de, no sé cuando tienen relaciones, ¿usted piensa que…?**

E: No tenemos relaciones

**P: No no… entonces vale. Y por ejemplo ¿nota si desde que toma la medicación ha bajado incluso más su apetito sexual? Desde la medicación, ya hará mucho tiempo ¿¿?¿?¿**

E: No, a ella lo que le baja el apetito sexual es lo que te digo como el dolor de muelas

**P: Ya. Que es un dolor generalizado.**

E: Es un dolor generalizado

**P: Claro**

E: Cuando… ahora, bueno es que se da la casuística de que tiene lo que es la operación… ¿¿?¿

**P: Claro**

E: Habrá que valorar el tema ¿¿?¿?. Cuando estás en pareja, estas a gusto P. Entonces, yo lo comprendo, que no puedes estar mirando todo el rato cama cama cama…

**(10:04)P: Claro**

E: A ver si me entiendes, que esto tenemos tiempo por delante. Y yo lo comprendo de que la persona que ha confiado en mí que al final el tema este sexual sea el último, pero el último, el último, el último de su lista.

**P: Claro. Hombre, en estudios como este por ejemplo sirven para también un poco el quizás darle la importancia que tiene la sexualidad. Y muchas veces, aunque sea lo último, pero y si lo ponemos un poquito antes quizás ayuda también. Que es muy difícil según el grado, según si hay crisis, según si no. Pero muchas veces nos olvidamos demasiado de la sexualidad, y bueno, según…**

E: Nosotros pienso… Fíjate lo que te voy a decir yo. La verdad que yo soy muy optimista con el tema… soy con un pensamiento muy optimista. Yo a mi mujer le digo, mira la asociación de fibromialgia tenía que estar en hermandad con la asociación de maridos de ninfómanas

**P: jajaja**

E: Y así, todos felices.

**(11:05) P: Todos contentos. Jajaja. Pues sí que hay que tener sentido del humor. Hay que ser positivo. Algún día jajaja**

E: Algún día, algún día jajaja

**P: No, pero sobre todo quizás, puede que algún día sí haya una medicación o haya un tratamiento o haya algo que pueda hacer que esto remita de alguna manera.**

E: Yo pienso que es el dolor. Te vuelvo a decir, es que siempre sigo con esto, es el dolor de muelas. Si yo tengo un dolor de muelas, si a mí me duele lo que es el cuerpo, si yo tengo mi pareja digo “pobrecillo que está ahí que si tal, que si cual… Fíjate todo el tiempo que hace, que cojo y me pongo”. Pero P, el tema de la sexualidad ha cambiado, vamos, ya no es como antes: “cojo pum pum el coito y san se acabó”. Nosotros por ejemplo, queremos algo más, yo por lo menos, entonces no es… Entonces por eso es.

**(12:03) P: Sí se complica más la cosa. Quizás el crear un ambiente, el estar como más…**

E: Lo que es una relación

**P: …como más en pareja de verdad, como más calidad y menos…**

E: Yo no sé lo que… Yo tengo un concepto de sexualidad que no es precisamente el coito.

**(12:23) P: Claro. No, pues mucho más sano porque eso es lo que muchas veces se evita. El que la gente…**

E: Yo con mi mujer en concreto, antes de, le decía “pero no, digo que si es que oye que si es que… “, a ver si me entiendes, yo disfruto más con… me acuerdo... Pues cuando ella no tenía lo que es el problema este, pues las noches que nos tirábamos horas y horas y horas y… más panchos que largos, y si había coito había coito y si no había coito pues no había coito, pero… siempre había coito jajaja

**(13:00) P: jajaj. Pero ya de final.**

E: Pero no es una cosa prioritaria ni mucho menos

**P: Pues eso es una mentalidad estupenda. Partiendo de esa base que es tan buena, que es una buena base…**

E: Sí sí

**P: …se supone que es más sana. Al tener un contacto que va más allá del coito en sí y que es una cosa más íntima, más piel con piel, más…**

E: La sexualidad es eso.

**P: Claro. Eso hace que…**

E: Es como dices, piel con piel y alma con alma. Pero lo que pasa que las dos tienen que estar relajadas para llegar a eso.

**P: Y tienen que estar en una sintonía**

E: Y con la enfermedad esta es muy difícil.

**P: Claro. Y por ejemplo, cuando… masajes y cosas así, eso por ejemplo ¿hay como cierta receptividad o mejora?**

E: Pues mira masajes. Masajes… cuando yo le doy el masaje a mí mujer…

**P: Sí**

E: … pues me emociono.

**P: Claro**

E: Y luego… jaja

**(14:00) P: Y luego no hay nada que hacer**

E: Y no, porque no. Parece mentira que sabes cómo es esto y que sigas que si “pa cual…” ¿¿?¿ Pero que es algo como si estuviera casado con un mariquita pues no me pasaría esto, pero… jajaj

**P: Pero bueno, también está bien que ella se siga sintiendo deseado ¿no? Que por ese lado…**

E: No sé, yo la parte femenina no. Pero me imagino, me imagino de que sí. Vamos digo yo. Peor sería que no se sintiera, y no sintiera… vamos digo yo

**P: Claro.**

E: Lo que pasa que ese deseo muchas veces se convierte en frustración, pienso yo.

**P: Claro. Eso sí.**

E: Cuando ella piensa que no me da lo que… por ejemplo, ahora está, “te pasa algo, qué te pasa” y no es que me pase, es que ella leva dándole vueltas a la cabeza. Es que no me acuerdo de la última vez que tuvimos relaciones, es que no me acuerdo. Y entonces dice, si es que llevo ya, no sé, si llevaremos cerca del año o lo que sea…

**(14:58) P: Claro**

E: … Y ella pues va dándole. Y siempre “tienes otra mujer, y tal que si cual” No, porque entre otras cosas se me notaría en la cara jajaja

**P: jaja. Si hoy en día con las rutinas que tenemos hay muy poca gente que tiene tiempo para dos personas, es como “este no sé como lo hace”. Con lo complicado que es llevar a una persona para adelante, como para tener dos**

E: ¿¿?¿?

**P: Entonces es más tranquilidad. Y por ejemplo, ¿qué le recomendarías a una persona que su pareja acaba de tener un diagnóstico de fibromialgia? ¿Cómo, o qué recomendación le daría para afrontar este tsunami que se presenta aquí y que de repente revuelve todo y hasta que se asienta es complicado?**

E: Compresión. La palabra es compresión. Hay que tener compresión y olvidarse de todo lo anterior. Nosotros hemos perdido amistades, pero no es por nada, es porque no les podemos seguir lo que es el ritmo. Es decir, que si quedamos para irnos de acampada o irnos de esto, pues de acampada… A nosotros nos gustaba mucho el camping y hacía ya que no íbamos de camping años y años y años. Y la relación con la gente, pues no es lo mismo irte de acampada que verte cuando ¿¿?¿ y poco más. Entonces, las personas con fibromialgia lo único que tienen ganas es de estar tumbados en el sofá y poco más.

**(16:30) P: Claro**

E: Entonces pues, a poco que te descuides, las relaciones sociales… A mí por ejemplo, me puede mucho. Yo voy sólo, y parece que estoy separado, pero no es por nada, es porque ya con mi edad tengo lo que son unas cosas más o menos claras. Tengo las cosas más claras, más o menos claras. Paso olímpicamente, en su justa medida, del whatsapp, del facebook y del instagram.

**P: Claro**

E: Yo prefiero que “P vamos a quedar y tomamos un cafelillo”, “vale venga rápido”, media hora, pum pam. Con amigos, cara a cara “oye, ¿cómo estás?”, nada, con familiares, con amigos… Y entonces, pero siempre voy sólo. Pero porque me gusta lo que es el trato cara a cara y no perder lo que es la amistad.

**P: Claro. Entonces, por ejemplo, el cuidar las relaciones sociales aunque sea fuera de la pareja también es importante para…**

E: Sí, como no lo hagas

**P: … una red de apoyo, el tener ese apoyo de...**

E: como no lo hagas, no es por nada, es que te quedas totalmente aislado. Pero no es por nada, es porque en las relaciones sociales pasa como con el amor, es la llamita, pero es que hay que cuidarla siempre siempre siempre. Y lógicamente, también las amistades tienen que tener un poquito de compresión.

**P: Claro**

E: Un poquito de compresión, es decir, “no viene porque no puede o tal”. Y bueno, pues siempre ¿¿?¿?¿?¿?¿ Pues no se sabe si va a aportar o no va a aportar, seguramente no aporte.

**(18:18)P: Claro**

E: Es difícil.

**P: Es que es eso lo que vemos, que por ejemplo los maridos son un apoyo extraordinario y grandísimo. Pero claro, no sabíamos “¿y qué piensan los maridos?, y ¿cómo viven?, ¿cómo pueden ser ese apoyo tan bueno?**

E: No sé ni cómo lo hago yo.

**P: Claro, pero es que esto es un aporte buenísimo porque ya solamente cómo una persona, el testimonio de una persona, puede ayudar a otras y si recogemos varios testimonios esto puede ser de gran ayuda para personas que por ejemplo no saben cómo afrontar un diagnóstico de una enfermedad tan desconocida. Entonces, yo creo que, todo lo que has dicho es súper valioso.**

E: Es triste y es penoso y es lo que siempre se dice. Ahora por ejemplo, cierra el mayor laboratorio privado de renovables de toda España. Ya verás, todos los científicos a la calle, al callejón.

**(19:29) P: Ya**

E: Anoche jugó el Madrid contra Nápoles. Toda la gente sabe “esta noche juega…” pero los científicos, que es lo que importa, que es lo que tienen que estar pagando, no está pagado.

**P: Claro**

E: Yo, en cambio, se lo he dicho ¿¿¿??? Es decir, si yo de mi sueldo tengo que poner todos los meses 100€ para pagar lo que es un científico en la universidad de Granada o tal, con la única… y te dicen “como te ha tocado a ti, pues es lo que te toca”. Pero claro, en plan egoísta también, es decir, si el tema no avanza. Pero son palos de ciego, todas las personas que tienen una enfermedad darían lo que fuera porque se recuperara.

**P: Sí, es verdad.**

E: Las personas que tienen fibromialgia tienen varias sensaciones de ansiedad.

**(20:27)P: Sí**

E: Una, “no puedo hacer lo que hacía antes”

**P: Claro**

E: Generalmente son, por lo que yo tengo vistos, en muchos casos, que solían ser personas hiperactivas. Dos, “tengo sensación de culpabilidad porque con mi familia, con mi pareja tal”. Tres, “si esto me pasa ahora con 50 años, ¿qué será cuando tenga 70?”.

**P: Claro.**

E: Entonces llevan lo que es…

**P: Es que eso pasa muchas veces, que cuando todos esos pensamientos se acumulan y no son capaces de, uno no es capaz de liberarse, eso incluso empeora. Porque muchas veces también, el cuerpo responde a cada emoción que sentimos y cada pensamiento nos lleva a una emoción. Entonces, si pensamos de una manera, ese pensamiento nos hace sentir de una manera, y esa emoción se queda en nuestro cuerpo como un sello por donde corresponda, porque muchas veces…**

E: Es lo que te digo, que la pareja es fundamental en el caso de… Porque si no hay momentos en los que yo he estado muy muy muy bajo de moral. Y bueno, que hay que ayudar en lo que se pueda.

**(21:47) P: Un apoyo increíble, la verdad. La verdad que siempre, el estudio que se hizo aquí hace un par de años, salió eso, que la pareja era fundamental.**

E: No participé yo en el estudio ya te he comentado.

**P: Sí, pero ella. Por eso, ahora queda ver el punto de vista de los hombres que… Por eso quizás nuestra entrevista es un poquito más larga.**

E: Esto P te lo digo a ti. Hay una mentalidad muy muy muy cerrada con respecto al tema este. Y yo por ejemplo tengo una mentalidad muy abierta con respecto al tema este. Y yo pienso que la sexualidad, es como el comer.

**P: Una necesidad básica.**

E: Entonces, desde el momento en que la sexualidad es como el comer, la pareja estamos, yo te digo como estoy con mi pareja, estamos chapados. Yo por ejemplo, agradecería que mi mujer me dijera “pues bueno, ¿necesitas echar un quiqui? Pues venga, ve y échalo que no pasa nada”.

**P: Ya**

E: Pues porque lo mismo que necesita ella ver un partido de fútbol o que necesita hacer lo que tal, que no necesita nada.

**P: Ya**

E: Sin embargo… pues no. Y yo eso, no lo veo. Por eso te digo

**(23:05) P: Claro es que eso no se ve por lo valores culturales que tenemos. Pero en realidad, es exactamente lo que usted dice, que muchas veces por qué vemos la sexualidad tan diferente, cuando en realidad podría ser mucho más fácil.**

E: Yo, yo no lo veo. Yo por ejemplo hablo con los amigos y me dicen tonto. Pero a ver si me entiendes, “¿quién se va a enterar? Tú, tú o tú”. También yo tengo la mentalidad antigua.

**P: Es la mezcla entre una cosa antigua y una nueva.**

E: Si yo por qué voy a coger… Si yo a mi pareja se lo digo todo, es decir, por qué voy a…

**P: a ocultar algo**

E: …a ocultar algo.

**P: Claro**

E: Pero agradecería por parte de ella, lo que pasa es que… ¡no!

**P: Claro, es que ¿cómo se abarca ese tema, cómo se aborda ese tema? Es como decir, ¿y yo cómo propongo esto?**

E: Porque entonces, ella se siente muy muy muy inferior, pero…

**P: Ya.**

E: Es que yo lo veo como el comer, a ver si me entiendes. Antes de que le diera, por ejemplo, lo que es el… nuestra actividad era muy buena, muy buena. Pero…

**(24:14)P: Y ahora claro, es que es eso. Es que por un lado está lo cultural que se tiene como la pareja y todo lo tiene que dar una persona y tal, y luego por ejemplo…**

E: Yo pienso que la asociación de fibromialgia tendría que enfocar a las pacientes al tema este que te estoy diciendo.

**P: (asiente).**

E: Es decir, “a ver oye, que no pasa nada”. A ver si me entiendes.” Que no se va ir con fulanita, ni con otra persona, que te quiere a ti. Y ya está. Pero que él tiene unas necesidades y lo tienes que comprender”

**P: Claro**

E: Es difícil. Es decir, que yo también me pongo en el lado contrario.

**P: Claro**

E: Hombre, qué bonito ¿sabes? Porque tengo una disfunción eréctil por ponerte un ejemplo, pues se va con… Pues es difícil. Pero en mi mentalidad, yo en el otro caso, también lo vería, a ver si me entiendes. Pero es por el tipo de mentalidad tan abierta, o abierta… o distinta que yo puedo tener.

**P: Claro**

E: Dentro de mi casuística.

**P: ¿Qué perdón?**

E: Dentro de mi casuística digo

**(25:21)P: Claro, claro, no si, pero que no es errónea. Yo creo que muchas veces como está vinculado también con tradición; tanto el sexo como el matrimonio pues de ahí no se sale. Luego hay nuevas tendencias que están diciendo pues incluso poliamor…**

E: P, que hasta ahora estamos hablando de un tema excepcional. Entonces, vamos a ver. “¿Tú tienes alguna duda, te pasa alguna duda por la cabeza, algo de que yo no te quiera a ti, de que no te ame a ti?”

**P: Ya**

E: Pues lo mismo, en el momento en el que no se habla de cocinar no pasa nada, porque vayamos a la tienda a comprarnos la comida, pues si hay esta carencia en la pareja, pues se sustituye por esto. Práctica, soy una persona práctica. Pero…

**P: Claro**

E: Eso ya es casuística de pareja. Pero me imagino que tratando de la enfermedad que estamos tratando pues se plantearán también en otros casos, ¡no seré yo el único!

**P: No, se plantearán y en otras patologías, por ejemplo en minusvalías de cierto tipo, también se plantean alternativas. Pero eso sería una cosa muy bonita de estudiar también, el decir cómo plantear en una asociación este tipo de alternativas para cuidar a la pareja. Es que es eso…**

E: Yo eso digo, lo que te he dicho antes, asociación de maridos de ninfómanas…

**(26:45)P: Claro**

E: Mira todo eso resuelto. Esos maridos “tranquilicos” el tener ahí…

**P: “Ay, que me la quito del medio un rato”**

E: Ya ves.

**P: No, pero es verdad, que muchas veces se olvida igual que por ejemplo las cuidadoras se dice que tienen el síndrome del cuidador ¿no? Una fatiga, una tal… ¿qué pasa con las personas que conviven con una persona que tienen una patología como la fibromialgia? Que si la enfermedad es desconocida, más desconocemos cómo lo pueden estar pasando esas parejas**

E: Pues yo me imagino que todos lo hablarán?¿?¿… no he comentado tema relación con mi mujer, en concreto¿¿?¿?¿? Cuando supe que tenía fibromialgia, antes de que cayera en un pozo … ¿¿?¿

**P: Estupendo, sí**

E: Entonces le apunté aquí en la asociación, y por lo menos… ¿¿?¿Pero yo con los otros maridos no he comentado nada, pero me imagino que… ¿sabes lo que pasa? Que por mi casuística, tengo mentalidad abierta, pero en otras cosas soy incapaz de engañar.

**(27:53)P: Claro**

E: y… entonces se lo digo y ella lo ve mal, pues nos aguantamos.

**P: Claro, claro. Es que es una cosa complicada, es como… ¿qué se hace aquí?**

E: (asiente)

**P: Pues si se podría, no sé en otras patologías se presenta esa alternativa, pero…**

E: Yo pienso P, a ver si me entiendes, que estamos en el siglo XXI…

**P: (asiente)**

E: que lo mismo que la juventud, una persona de 20 años no tiene nada que ver con una persona de 20 años de hace 20 años, no tiene nada que ver.

**P: Nada**

E: Pues… sin molestar a nadie, sin molestar a nadie, sí podríamos ir rompiendo tabús y dando soluciones a algunos temas.

**P: Pues sí.**

E: ¿Por qué no?

**(28:51) P: Pues si buscando información, encuentro algo relacionado con eso, ya a nivel personal, le puedo incluso mandar un email con algún artículo de alguna…**

E: A ver si me entiendes. Yo me estoy sincerando contigo…

**P: Sí sí sí, yo para ayudarle**

E: ¿¿?¿? al estudio de… que no te echen un ave maría purísima

**P: Digo sobre todo por ayudar…**

E: Ayuda tiene que ser función de…

**(29:25) P: de la asociación**

E: … de la asociación, de la psicóloga que vayan abriendo la cabeza, es decir, “oye, que no pasa nada, que esto es lo que hay, y vamos a estar aquí en 2 días y lo mismo que vosotras tomáis lo que son las pastillas, también tenéis que mirar por vuestras parejas y tratarlas”. Es que eso es así. Y nada más

**P: Pues sí. Pues si quieres lo podemos dejar aquí, que ya tenemos todas las preguntas…**

E: ¿Ah sí? ¡Pues estupendo!

**P: Sí. Porque si acaso, es que como se han respondido algunas preguntas a medida que íbamos hablando, como por ejemplo, en tareas del hogar y eso, pues que más o menos se hacen…**

E: ¿Tareas del hogar?

**P: Sí, ¿cómo se llevan? ¿Cómo pueden compartir tarea?**

E: Sí, las tareas, lógicamente hay que echar una mano cuando. Te digo también, yo tengo una mentalidad pues todavía machista. Machista digamos, en mi casa mi padre no ha hecho absolutamente nada.

**(30:33)P: Claro**

E: Nada absolutamente. Eso ha cambiado con el tema, pero yo pienso que le podría ayudar mucho más.

**P: Sí.**

E: Lo que pasa es que yo por ejemplo tengo otra mentalidad totalmente diferente a mi mujer.

**P: (asiente)**

E: Yo por ejemplo cuando dan las 11 de la noche suelto la escoba y “pam” aquí me ha llegado.

**P: Claro**

E: Se cae ahí y echo la puerta de mi casa, y mañana más. Porque ahora quiero ponerme el pijama, sentarme en el sofá, cogerme la tablet y tal. “Pero es que hace falta hacer de esto y de esto”, “mañana, mañana”.

**(31:02) P: Claro. Hay ciertos límites para las tareas**

E: Claro, es lo que te digo. De toda la vida a ella le han dado las 3-4 de la mañana y está haciendo cosas, y yo no.

**P: Claro.**

E: Yo digo “necesito un tiempo para mí” Cuando digo tiempo para mí, es media hora, tres cuartos de hora, pues yo cojo “pum, ¿qué me gusta?” Me gusta leer un artículo del periódico, cojo y lo leo. Y si a lo mejor tengo los platos por fregar, pues muy bien, mañana.

**P: Ahí se pueden quedar, que de ahí no se mueven nunca. Es verdad**

E: No puede ser que me lleve una sorpresa y que mañana cuando venga alguien los haya lavado

**P: Ojalá. No, pero eso está muy bien. El decir, “pues, mi tiempo para mí”, y es muy sano.**

E: P

**P: Mis 8h de sueño y mi… estar tranquilo**

E: Si tú te levantas a las 6:30 de la mañana y empiezas pum pum pum con la dinámica del día, que tal que si cual, que si luego los niños, que si luego los padres, que si tal, que si pa’arriba, pa’abajo… y tienes que decir “he llegado hasta aquí, y ahora bien, media hora para mí”

**P: Pues sí**

E: Estoy pidiendo media hora de 24h, pues tampoco es tanto

**(32:11) P: Sí, para relajar, respirar, leer un articulito y prepararse para la cama. Y luego por ejemplo, si acaso lo último, a partir de la medicación, ¿ha habido un cambio corporal grande, por ejemplo a nivel que ella se haya notado como quizás más decaída, más hinchada, más…?**

E: Mi mujer tiene lo que es la fibromialgia, tiene un 85%, en el estudio que le hicieron en la Universidad de Granada, tiene 85% de fibromialgia, tiene fatiga crónica. Y si tú vieras la medicación que se toma todos los días, te quedarías pasmada, es impresionante. No me acuerdo ya P. Te digo que hace ya más de 10 años, y lo único que me acuerdo es, rememoro, las relaciones de pareja que había antes y rememoro, que ella ha sido una persona que ¿¿?¿ Yo de toda la vida de Dios he sido igual, es decir, yo llegaban las 11 de la noche y dejaba?¿?¿ la escoba fuera, pero ella no, ella seguía y seguía y seguía. Dándole machaque, “que me puedes ayudar, que si cual”, “ha llegado la hora y he soltado la escoba”. Pero seguía. Pero ya está. Es lo único.

**P: Pues yo creo que ya está. Porque ya está todo respondido.**

E: Muchas gracias P.

**P: Muchísimas gracias**

**P – ¿Hace cuánto tiempo le diagnosticaron fibromialgia a su mujer, más o menos?**

M – Ella tiene problemas de…Yo creo que ella lo sospechaba hace tiempo. Lo sospechábamos desde hace tiempo y, además, sin conocerlo muy bien. De decir, tú tienes algo que no es, porque… Tuvo una necrosis en la muñeca y por eso es por lo que tuvo que dejar la peluquería. Y, y, problemas. La espalda, eh, con la espalda tiene problemas. Tenía, tiene una hernia de disco y demás. Y tenía un problema de tipo de, me duele aquí, me duele allí, de tal… Entonces, desde hace bastante tiempo. Pero, pero diagnosticado, yo diría que desde hace 3, 4 años. No sé muy bien. Tengo memoria, cronológica, tengo… Soy bastante malo.

**P – Sí, es difícil muchas veces.**

M – Pero, pero creo, que diagnosticado, de decir, tengo esto… Afrontado, menos.

**P – Claro.**

M – Ella no lo… Por su forma de ser, no lo, no lo quería exteriorizar. No lo tenía y tal. Es como si lo tuviera que tener callado y tal. Afrontarlo, menos. ¡Qué va! Afrontándolo, mucho me… muy poco. Dicien… Diciéndolo abiertamente, tengo esto. Pero vamos, yo diría que 3, 4 años.

**P - ¿Y desde entonces cómo ha cambiado, por ejemplo, la vida en casa? Eh… Desde el diagnóstico, al ponerle un nombre y eso, ¿cómo? Luego, por ejemplo, ¿cómo se da ese día a día? Porque, por ejemplo, muchos pacientes dicen que es como, como un *tsunami*, ¿no? Que llega de repente la noticia, tengo esto, y es como… todo cambia, y ahí, hasta que se, eh, recupera el equilibrio, que no va a ser igual que el de antes… ¿cómo afecta?**

M – Yo cre… Es que ha sido muy progresivo. Entonces, ha sido muy progresivo y, además, con etapas en las que tuvo que dejar Ø y con etapas anímicas, malas etapas anímicas. Lo pasamos muy mal, pero ya no. No ya por lo, por lo, por los síntomas, de dolores y demás, sino que laboralmente le afectó mucho. Una persona que lleva desde hace mucho, que empezó desde muy joven a ser independiente. Laboralmente, laboralmente y en todos los sentidos, ¿no? Y entonces, le ha afectado mucho anímicamente, es decir, el verse, vamos a decirlo “indefensa”, a lo mejor no es la mejor expresión, ¿no?, pero el verse, el no ser capaz de, de ser, de trabajar, de ser capaz en todos los sentidos, eso yo creo que lo que peor lo ha llevado, en det… eh, eh, por rachas, más incluso que el dolor físico. Porque creo que Ø es bastante fuerte e, incluso, intenta no exteriorizar de, me duele esto, me duelo lo otro. Se le nota, ¿no? Logicamente.

**P – Sí.**

M – Pero que ¿cómo ha cambiado? Pues ha sido muy progresivo. No ha sido, no ha sido una Ø. Ni siquiera cuando le, cuando el diagnóstico era claro.

**P – Sí.**

M – Es decir, tienes esto, tienes lo otro, punto, y demás y tal y tal y fibromialgia. Y salió en el papelito, en el papelito fibromialgia. No fue una sorpre… Al menos a mi me lo pareció. Para mí no fue una sorpresa. Quizás para ella sí y no, y no lo exteriorizó.

**P – Claro.**

M – Es como si ya se sospechara que había algo, había algo ahí y que, y que iba, no sé… Incluso, casi, si me apuras, el hecho de que fuera fibromialgia… A lo mejor es muy fuerte esto que te digo, ¿no?, pero es casi un alivio, porque decir, es fibromialgia, ya sabemos lo que es, Ø, pero no es nada más grave que se puede esperar. Porque ella ha tenido, ella tuvo episo… Bueno, ya te digo, una necrosis en un huesecito, una tontería. Vamos, una tontería, no. No una tontería, pero le impidió trabajar. Entonces, claro, el hecho de que esto pudiera ser y tal y las articulaciones y tal, y eh, eh, podíamos sospechar, o podía ella pensar, con poca comunicación en ese sentido, porque no le gusta hablar de eso, aparte de que no le, sus dolencias no las exterioriza. Últimamente, más, pero intentaba hacerse la fuerte y demás, y tal. Entonces, ya te digo, que, que… Fue muy progresivo. No hubo una ruptura de decir aquí pone fibromialgia, ¡oé, qué papelón!, ¿no? Como si ya estaba en el aire, estaba en el ambiente de, de antes de que lo pusiera el papelito.

**P – Claro, por el tipo de síntomas y por…**

M – Sí, efectivamente.

**P – Y, por ejemplo, eh, ¿cómo se reparten las tareas en el hogar? ¿Hay como un… se reparte más o menos equitativo en plan de… o hace ella mucho?**

M – Ella hace más que yo. Más que yo. Hay un pacto. No está escrito, ni… casi ni hablado, ¿no? Pero por esto digo, hace bastante tiempo en que ella, digamos que tiene más tiempo, ha estado un tiempo en paro. Entonces, el hecho de que lo haga… Además, le gusta cocinar. SE mete en la cocina… ¡Este es mi sitio! No porque… Lógicamente… A mí, entre comillas, bueno, esto no es políticamente correcto, pero, si tú te encargas de eso, bueno, vale, ¿no?

**P – Claro, un reparto, ¿no? Un…**

M – Sí, bueno, un pacto. Yo lo entiendo así. Que tampoco tiene que ser al 50%. Lo que tiene que haber es un acuerdo, y un acuerdo amistoso y armónico y demás. Y yo, pues a lo mejor me encargo de menos, cosas, pero más feillas o yo que sé. Llevar a la niña para allá o con la niña, que la tenemos pequeña, que tiene 11 años. Pues cosas que, que, pues eso, menos cosas domésticas, pero a lo mejor, las más feas o las que menos…

**P – Sí, más engorrosas.**

M – Las más… o las que menos le apetece a ella…

**P – Claro.**

M - ¿No? Es decir, oye, tú te encargas de esto y tal. Bueno, a lo mejor, pues yo siempre he pensado: ¡Ah, bueno, pues salgo ganando! Porque, a lo mejor para ti es feo, ¿no?, pero a mí, pues, no me importa mucho.

**P – Claro, eso es lo ideal.**

M – Ø ¿Y yo me encargo de la niña? ¡Venga, vale! Pero ponme un café.

**P – Claro.**

M – Igual que el chiste famoso de la basura, ¿no? Que no se enteren las mujeres que nos gusta sacar la basura, ¿no? ¿Por qué a las mujeres no les gusta…? Vamos, dicen. Y parece ser que es así. Les horroriza salir a la calle sin arreglar y demás… ¿para tirar la basura? No lo sé. A lo mejor es un poco esto. Pero una decían dos, oye, que se enteren estas que nos gusta tirar la basura. Pero claro, a lo mejor hago yo más cosas… Bájate tú a tirarla. Y por eso es una broma, pero vamos, un poco por ahí…

**P – Sí.**

M – En ese sentido, sí. Ella trabaja más que yo en casa.

**P – Y, por ejemplo, ¿ella está tomando medicación ahora mismo?**

M – Sí, mucha.

**P – Mucha. Y ¿cómo le afecta la medicación? ¿Le ayuda?**

M – Para bien.

**P - ¿Para bien?**

M – Sí. Yo creo que ha tenido, porque ha tenido… Bueno, últimamente, ha… Uno de los medicamentos, o un par de los medicamentos, ha descubierto, que no sé si es por la humedad o porque ahora se le ha, le ha brotado un, una, ¿una?... ¿alergia?

**P - ¿Qué no es compatible?**

M - ¿No es alergia?

**P - ¿Una intolerancia?**

M – Sí, intolerancia. Bueno, intolerancia o alergia. Una de ellas es alergia. Entonces, le ha sentado bastante mal y ha estado bastante fastidiadilla con el estómago y con síntomas feos y demás. Pero parece ser que era alergia y… En general la toma de medicamentos, no le, no l ha sentado mal en sí mismo. Lo que no le ha sentado bien es, es una combinación de medicamentos que le han dado. Unas veces ha ido bien. Ahora, creo, que va bastante, creo que va bastante bien. Pero sí. Porque, además, se está tratando también para… Tiene antidepresivos y demás. Ø y Diazepan, entre otros. Y… indepedientemente de, creo que le vienen bien, estando mucho mejor de ánimos y demás. El, los analgésicos también le han venido bien hasta ahora, y yo creo que hay una cosa muy importante, que no se siente drogada, ¿entiendes? ¿Sabes la, la sensación esa de decir, estoy drogada, me tienen empastillada, estoy drogada y demás? Y creo que tiene… El médico de cabecera nuestro… Es de ella, perdón. Es muy amigo, es amigo suyo. Habla mucho con él. Es muy buen médico y habla mucho con ella. Y, entonces, un poco como que confía en él, como decir, oye, mira, que me Ø pastillas y estoy…

**P – Es muy importante que el médico… Que uno confíe en el médico.**

M – Claro, claro, porque sé que lo que quiere es eso. No quiere quitarlo de en medio. No quiere, simplemente, drogarme, entre comillas, o dormirme, o que esté ahí tal. Y en ese sentido, creo que eso, que no se siente, que no se siente manipulada Ø

**P – Que ella es parte del tratamiento.**

M – Ha asumido que se tiene que medicar y ahora, últimamente, creo, que le, que le va ben.

**P – Que bien. Claro, porque hay, a muchas paciente, según qué medicación, reaccionan en plan de… Por ejemplo, se sienten muy hinchadas o, eh, por ejemplo, bueno, y eso hace que se baje un poco la autoestima, y esto también interfiere a, en la relación de pareja y…**

M – Sí, está claro. Asume que la medicación que está tomando, ha estado tomando y demás Ø La libido, dice… porque además se lo ha explicado perfectamente, y lo asume, y lo asumimos, claro, los dos juntos. (aprox. Min. 10)

**P - ¿Y cómo afecta a la pareja el, esta bajada de libido, por ejemplo?**

M- ¿A la pareja?

**P – Sí.**

M – No nos ha afectado mucho. Ahora, en cuestiones sexuales, un disparate, un disparate, un disparate. Tenemos muy, muy pocas relaciones sexuales. A la pareja, a mi particularmente, no me ha afectado. Es decir, lo he asumido. Lo he asumido y bueno, y ya está. Y, valoro, valoro la relación más que esto, que es importante…

**P – No, eso es importante. Y, por ejemplo, a nivel de relaciones sexuales, eh, por ejemplo, ¿han encontrado alguna alternativa de, que no sea…? Por ejemplo, nuestra sociedad hoy en día es muy coitocentrista y todo está como muy pensado hacia el coito y nada más, y, por ejemplo, hay alternativas, pues no sé, quizás un masaje, u otro tipo de contacto piel con piel que no sea tan así… Que quizás para personas que tienen fibromialgia puede ser un poco más…**

M – llevadero…

**P – Claro. ¿Han intentado alguna alternativa?**

M – Tiende, tiende a ser cada vez menos frecuente. Una ra… ha, hay una razón también añadida ahí, y es que el orgasmo a ella le provoca un dolor en la pierna, un, un dolor…

**P - ¿Un reflejo nervioso?**

M – Un dolor, Un dolor que, además, está todavía ilocalizado. Empezamos a localizarlo… Ella empezó a localizarlo justo, justo en ese momento. Es decir, después del orgasmo ella se tiene que tomar un, un paraceta… un ibuprofeno. Es rarito, pero es así. Así pasa. Entonces, claro, el iniciar unos “pre”, y, y, y no llegar ahí, pues… Y no llegar o llegar con dolor… (min. 12 aprox.)

**P – Claro, es que eso es una faena.**

M – Es que, es que, claro, es que yo, o lo noto. Eso lo noto. Claro, y esto también me hace a mí, que entiendo que el juego sexual o el orgasmo en el hombre es distinto que en la mujer, vamos, entiendo no, lo sé, ¿no? Entonces, es mucho más de… ¿no? Ella, a lo mejor, es capaz de decir hasta aquí llego, ¿no? Yo soy capaz de decir, pero sufriendo. Por lo tanto, tiendo a no iniciarlo, tiendo… Tiendo no significa que no, que renuncie, y, de hecho, yo sé que a ella le cuesta un esfuerzo. Primero, por la medicación y ese tipo de cosas, y segundo, porque su final es cero.

**P – Hombre, es que si está asociado a un dolor…**

M – Directamente, pero automático.

**P – ES que eso es complicado.**

M – Es automático. Es decir, le dura 2 minutos. En 2 minutos vienen dolores en la pierna, que yo entiendo que…

**P - Pero, ¿Cómo ciática o como…?**

M – Algo así, pero no estoy muy seguro porque no lo he sufrido yo. Pero, pero sí, como… Sí, como ciática. (minuto 13.30 aprox.)

**P – Y, por ejemplo, ¿han ido a un especialista o algo?**

M – A mil.

**P - ¿Para el dolor ese? Y no…**

M – De todo tipo. Bueno, ya te lo explicará ella ahora tranquilamente. De todo tipo. Desde ginecólogo, Ø, tu-tu-tu, traumatológico.

**P - ¿Y nadie sabe de qué, a qué es debido?**

M – No.

**P – Jolín, es que nunca he escuchado nada así, pero vamos…**

M – Ahora te lo va a contar ella con detalle seguramente.

**P – Claro, que quizás…**

M – Entonces, eso condiciona, eso condiciona.

**P – Hombre, un condicionamiento clásico.**

M – Bestial. Es de forma bestial, y…

**P – Me puedo imaginar.**

M – Y… eso te digo, en las relaciones sexuales. En las relaciones lo hemos asumido perfectamente y, vamos, yo, por lo que me toca, lo he asumido perfectamente y, por decirlo de una forma rápida, eh, he renunciado. No lo voy a decir con agrado, porque no es así, pero he renunciado de buen grado a que tengamos unas relaciones… Hombre, no, a ver… De vez en cuando, pues nos damos unos Ø, un homenaje, como decíamos aquel y tal, aún a pesar de eso, o… bueno, pero que… (minuto 14.50 aproximadamente)

**P – Que se complica la cosa.**

M – Hum…

**P – Claro, es que lo de… muchas veces hay personas que, bueno, tienen una libido más baja y tal, pero claro, o que los dolores simplemente tampoco… (min. 15) Se limita muchísimo el tema de las relaciones sexuales, pero claro, ella al tener dolor asociado, que viene justo después… Y me puedo imaginar que debe ser… ¡Qué faena! ¡Qué…! Y, por ejemplo, eh, bueno, ya me ha dicho que eso no interfiere en las relaciones de pareja a nivel de comunicación o…**

M – No interfiere al 100% no es la palabra clara tampoco. Pero lo que hay… Vamos a ver… Interfiere. No es motivo de disputas, no es motivo de disgustos, no es motivo de, de, de discusiones, Ø caso. Que interfiere, pues estoy seguro… Si tuviéramos una relación sexual, eh, normal, pues sería muy distinto a lo que tenemos. Pero me refiero a que no interfiere… (min. 16) A lo mejor en algún momento sí. En algún momento, porque nos ha pillado, a cada uno le pilla en un momento. Bueno, a mí me pilla pensando en esto, a ella le pilla que en algún momento determinado del es, de su vida, de sus dolores, o de lo que sea, ¿no? Y entonces, pues bueno, pues un poco estamos las cosas así, Ø ha habido algún momento de discusión o de no quiero saber nada de esto y demás, pero vamos, muy puntualmente. En general, lo hemos, con el tiempo lo hemos ido asumiendo. Con el tiempo y con la evolución de, de, con la evolución o con el descubrimiento de la enfermedad y de sus propias dolencias. Porque, además, tiene asociado, pues eso, algo que no es de fibromialgia, sino que ya tenía de antes y demás. Un poco que se ha sumado a la fiesta.

**P - ¿Y cómo es la comunicación entre ustedes? ¿Es una comunicación abierta y se comprenden y tal, o…**

M – Se lo, sip… (min. 17) Por mi parte, yo tengo que decir que sí comprendo. La comunicación no es expresa abiertamente. Es decir, no hablamos abiertamente, pero entiendo que sí que nos comunicamos, que hay comunicación o que hay entendimiento.

**P – Ø mucho mejor…**

M – Un entendimiento tácito, es decir, Ø lo otro y nos entendemos. Discusiones, obviamente, de vez en cuando. Luego, en épocas en las que ella estaba mal o que no estaba debidamente, con la medicación adecuada, o que ella se encontraba, una época en la que ella se encontraba muy mal… Depresión directamente. Ø el ombligo, tu-tu-tu. Ø Y ese tipo de cosas, ¿no?

**P – Claro, es que ese proceso de adaptación hasta que ella ha podido ver que, pues aceptar el…**

M – No importa

**P – Es importantísimo, claro**

M – Ø abierta, abiertamente Ø Y ahora mismo está de, dentro de lo que se puede esperar, Ø al menos…

**P – Sí, que está bastante mejor, ¿no? ¡Qué bien! Y, por ejemplo, eh, por ejemplo, si una persona, a un hombre le acaban de decir que su pareja tiene fibromialgia, ¿qué consejo le podría dar para afrontar esta nueva situación? (min 18.30)**

M – ¿Yo, como pareja de alguien que tiene fibromialgia a otro hom… a otra pareja de otra persona, Ø porque este es de la mujer?

**P – Sí, fibromialgia… Si, sí, como de hombre a hombre… Solamente un 1% de los hombres…**

M – ¿De los hombres, no?

**P – Sí. Hay, hay fibromialgia. Por ejemplo, decían que era entre 1/8 y 1/20. De cada 20 mujeres, 1 hombre tiene fibromialgia; de cada 8 mujeres, un hombre tiene fibromialgia. Pero la pregunta es fácil, pero es difícil de formular… Es como, ¿qué consejo le daría a una persona…**

M – ¿A una pareja ajena?

**P – Sí, sobre todo, o a la pareja o solamente a él. Lo que quiera.**

M – Es difícil.

**P – Es que es complicado.**

M – Es difícil porque, porque yo entiendo que… Hombre, lo fácil sería decir, hazlo como yo, que creo que lo llevo bien, que… Pero, ¿qué he hecho yo? Pues no lo sé. A lo mejor es, quizás, por mi forma de ser. Entonces, hay, hay, yo creo que hay algo que ha influido bastante. Nosotros, a pesar de que nos conocemos desde hace mucho tiempo, yo soy divorciado, y nos vimos después de mucho tiempo. Y nuestra relación empezó siendo ya maduros. Entonces, po… La niña vino, pues cuando teníamos 40, y nos reencontramos 3 o 4 años antes. Es decir, nuestra relación no es veinteañeros, treintañeros, ni siquiera eso… Entonces, yo ya venía de mi historial. Es decir, he vivido, he hecho, he tenido relaciones sexuales, las que he podido… he querido. Las que he podido… Ella, igualmente, entiendo yo. Entonces, digamos que ya vas, casi, casi, que lo que tenías para aquella época, lo que tenía que pasar ya lo tengo. Es decir, yo entiendo que es distinto decírselo a un chaval con 20-30 años, que diga, oye, que tienes que renunciar a tu vida sexual… ufff… Eso me lo dicen a mí con 20-30 años y… Eso es una losa… Te lo dicen con 40, después de ya cierto recorrido y, dices tú, bueno, pues a lo mejor lo que me toca ahora es tranquilizarme, ¿no? Ø usted tenía un coche que corría mucho, pues ahora no, Ø uno que corra un poco menos, pues ya está, ¿me explico? Ø lo mejor, lo mejor símil, es decir… Eso estoy seguro de que me ha ayudado. Es decir, yo ya, yo tengo Ø y entonces, bueno, pues, uh, ahora toca estar como estoy Ø Por eso te decía, Ø ¿cómo le digo yo esto a una pareja? Pues depende de la edad. A un chaval de 20 años, decir, haz lo mismo que he hecho yo, no, porque es casi decirle, te sacrificas toda tu vida, o hipoteca toda tu vida a tener una relación sexual… casi nula no, pero… al 10% o al 20%. Es terrible. Ahora, si eso se lo tengo que decir a un hombre con 60 años, digo yo, mira, que esto es lo que hay y no te va a costar mucho trabajo. Es, dependería de eso. Y, en todo, paciencia. ¿Qué le vas a hacer? ¿Vas a Ø la relación? Y si la balanza te sale muy positiva Ø (min. 22)

[Interrupción]
